# Exceptional warming over the Barents area

**DOI:** 10.1038/s41598-022-13568-5

**Published:** 2022-06-15

**Authors:** Ketil Isaksen, Øyvind Nordli, Boris Ivanov, Morten A. Ø. Køltzow, Signe Aaboe, Herdis M. Gjelten, Abdelkader Mezghani, Steinar Eastwood, Eirik Førland, Rasmus E. Benestad, Inger Hanssen-Bauer, Ragnar Brækkan, Pavel Sviashchennikov, Valery Demin, Anastasiia Revina, Tatiana Karandasheva

**Affiliations:** 1 Norwegian Meteorological Institute, 0313 Oslo, Norway; 2 Arctic and Antarctic Research Institute, St. Petersburg, 199397 Russia; 3 Saint-Petersburg State University, St. Petersburg, 199034 Russia; 4 Polar Geophysical Institute, Apatity, 184209 Russia

**Keywords:** Climate sciences, Atmospheric science, Climate change, Cryospheric science

## Abstract

In recent decades, surface air temperature (SAT) data from Global reanalyses points to maximum warming over the northern Barents area. However, a scarcity of observations hampers the confidence of reanalyses in this Arctic hotspot region. Here, we study the warming over the past 20–40 years based on new available SAT observations and a quality controlled comprehensive SAT dataset from the northern archipelagos in the Barents Sea. We identify a statistically significant record-high annual warming of up to 2.7 °C per decade, with a maximum in autumn of up to 4.0 °C per decade. Our results are compared with the most recent global and Arctic regional reanalysis data sets, as well as remote sensing data records of sea ice concentration (SIC), sea surface temperature (SST) and high-resolution ice charts. The warming pattern is primarily consistent with reductions in sea ice cover and confirms the general spatial and temporal patterns represented by reanalyses. However, our findings suggest even a stronger rate of warming and SIC-SAT relation than was known in this region until now.

## Introduction

Changes in surface air temperature (SAT) and sea ice are the main drivers of the ongoing environmental transformation of the Arctic^1^ and have emerged as a leading signal of the global warming^2,3^. For more than four decades, the Arctic sea ice extent has declined almost continuously, with the largest trends in September and the smallest in March^3^. Between 1979 and 2021, the September trend was − 13.4% per decade, while the March trend was − 2.6% per decade^4^. In addition, the sea ice trend is accelerating for all calendar months, meaning larger losses towards present time^5^.

The Arctic SAT for 2020 marks the 9th of the last ten years when SAT anomalies were at least 1 °C higher than the 1981–2010 average^6^. The Arctic climate is trending away from its 20th-century state and into an unprecedented state with accelerated warming since 2005^7^. The long-term Arctic instrumental SAT records show an annual warming rate that has increased from 0.3 °C per decade over the period 1951–2015 to 0.9 °C per decade over the period 1996–2015^8^. According to the fifth generation European Centre for Medium Range Weather Forecasts atmospheric reanalysis of the global climate (ECMWF-ERA5^9^), the Arctic SAT warming rate is about 0.6 °C per decade within the period from 1971 to 2019, which is three times as fast as the global average^7^.

**Figure 1:**
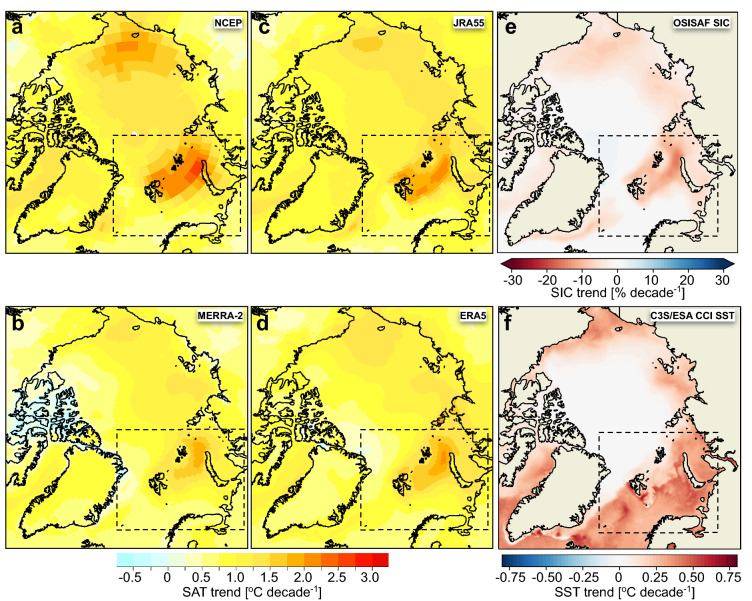
Spatial pattern of the Arctic warming and sea ice decline. (**a**)–(**d**) show trends in annual SAT (°C/decade) during the 1981–2020 period derived from various reanalyses sources (see Methods) that have been widely used for the Arctic: (**a**) NCEP-reanalysis, (**b**) MERRA-2, (**c**) JRA55 and d) ERA5. (**e**), (**f**) show annual trends in SIC (%/decade) and SST (°C/decade) (EUMETSAT OSI SAF, C3S/ESA SST CCI). The Barents study area is marked with dotted lines. We used the esd R-package (https://doi.org/10.5281/zenodo.29385) to create the maps in (**a**–**d**). The maps in (**e**–**f**) were generated using Python 3.6 (http://www.python.org) including pyresample 1.19 and cartopy 0.18.

Both the SAT analysis from instrumental records^8^ and widely used reanalyses products, including ERA5, point to a maximum warming area in the Barents region (Fig. 1). This Arctic warming hotspot^10^ is not constrained to the warming atmosphere; the Northern Barents Sea (NBS) region also hosts the most pronounced loss of Arctic winter sea ice^11^ and has since the early 2000s experienced a sharp increase in both temperature and salinity in the entire water column. The decline in the Barents sea ice cover, increased ocean temperature and salinity are closely related to the higher temperatures in the Atlantic Water and increased ocean heat transport entering the region from the west^12–14^. In addition, the increase in salinity is larger towards the upper layers, leading to a weakened ocean stratification and hereby an increased upward heat flux^10^. These oceanographic processes strongly contribute to the amplified warming in the region and enable larger heat flux interaction between the ocean and the air. If the rise in ocean temperature and salinity continues, the originally cold and stratified Arctic shelf region may be transformed into an Atlantic-dominated climate regime with a warmer and more well-mixed water column strongly preventing sea ice formation^10^. However, the Barents sea ice cover is largely affected by sea ice transported from the Arctic Ocean, and events of sudden enlarged sea ice or freshwater influx to the region may revert or postpone this Atlantification^14,15^.

Although the recent changes in ocean climate and sea ice in the NBS have been well documented in a number of publications in recent years^14,16–20^, there have been few studies on systematic changes in SAT based on instrumental observations, especially in the northern and eastern NBS. From long-term instrumental observation series high positive temperature trends were observed in the Svalbard region connected to the “early twentieth century warming”^21^, e.g. an annual warming during 1920–1942 of 0.3 °C per decade and a winter warming of 1.2 °C per decade^22^. However, these studies were limited to the western and southern part of the region. In more recent decades, various reanalyses and instrumental long-term series have shown distinct spatial differences in the SAT warming pattern within this region. Further, the most recent assessment on instrumental observations in the Arctic shows the largest temperature increase over western Svalbard^8^. However, this is based on a very limited data set for the NBS. On the other hand, various reanalyses that have been widely used for the Arctic (Fig. 1) indicate that the largest warming takes place near Franz Josef Land and Novaya Zemlya in the northeast^7,23^. Furthermore, the annual warming from ERA5 of 1.6 °C per decade in the northeast^7^ is significantly greater than 1.0 °C per decade over western Svalbard, seen in instrumental observations after they have been adjusted for the same period 1971–2019^8,24^. The scarcity of available near-surface in-situ observations in the region makes reanalyses more dependent on model assumptions compared to more data-rich regions, and it both hampers the validation and reduces the confidence of the reanalyses in this region. Recently, it has been shown that a warm bias is present at the surface and over sea ice in most reanalyses^25–30^.

On Svalbard, most of our knowledge about SAT development is based on long-term instrumental observation series limited to the western and southern part of Svalbard^22,24,31–36^. To our knowledge, the SAT development of northern and eastern Svalbard remains unexplored^34^.

In this study, we compile and analyze a large dataset on instrumental SAT observations from the archipelagos Svalbard and Franz Josef Land (FJL), located on the border between the Barents Sea and the Arctic Ocean (Fig. 1). This new dataset covers the period 1981–2020, has unprecedented spatial coverage, and provides time series that are longer than those that have been used by the scientific community so far. Indeed, some of the data series have not previously been available for scientific analysis. The quality of the SAT series has been improved further by extensive quality control and metadata from the archives. Here, they are compared with the SAT data from the most recent ECMWF reanalysis data set (ERA5^9^) and the recently released high-resolution Copernicus Arctic Regional ReAnalysis (CARRA^37^) from Copernicus Climate Change Service (C3S). The SAT trends have been evaluated against sea ice concentration (SIC) and sea surface temperature (SST) data from global data sets (EUMETSAT OSI SAF and ESA SST_cci, respectively) and high-resolution ice charts (MET Norway). Our main objectives are threefold: i) establish an extended and consistent high-quality SAT-dataset covering the NBS region, ii) study the recent warming and its spatial and temporal variability over the NBS, and iii) relate the trend in SAT pattern to variations in SIC and SST. Specifically, we address and discuss the following questions:

- How do the SAT trends in northern and eastern Svalbard and FJL compare to those in western and southern Svalbard?
- How well do reanalyses describe SAT-climatology and SAT trends in the Barents study area, especially for sites and periods without SAT observations available for assimilation?
- How much of the SAT variability is coupled with the SIC and SST variations?

We find an unprecedented increase in SAT over the NBS of up to 2.7 °C per decade annually at Karl XII-øya in northeastern Svalbard, with a maximum in autumn of up to 4.0 °C per decade. The warming is greater than hitherto known in this region and exceptional on the Arctic and global scale. We show that the warming is strongly linked, both in space and time, to the large reduction of sea ice and increased SST and that the observed temperature increase is in good agreement with the reanalysis. Our results additionally demonstrate that while CARRA and ERA5 reproduce the gross features of the observed trends, CARRA does it with more spatial details and larger regional SAT trends.

## Results

### Recent surface air temperature (SAT) development

#### SAT development from reanalyses

**Figure 2:**
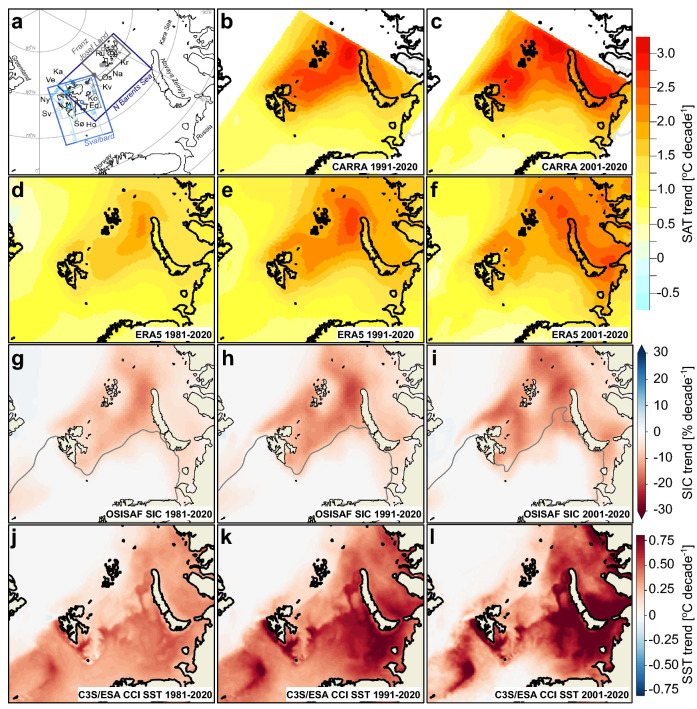
The spatial pattern of changes in surface air temperature, sea ice and sea surface temperature in the Barents study area for the time periods 1981–2020, 1991–2020 and 2001–2020. (**a**) Barents study area including the 13 weather stations which are shown with symbols and the first two letters of the station name from Fig. 3b (for larger map and more details, see Fig. S1). The regional boxes used for time series analyses (Table 1 and 3) are marked on the map, including the four Svalbard sub-regions. (**b**)**–**(**f**) Annual SAT trends (°C/decade) derived from CARRA and ERA5. Please note that the results are mapped onto different grid resolutions. (**g**)–(**i**) Annual trends in SIC (%/decade) with mean 15% SIC (ice edge) contour line marked in grey, and (**j**)–(**l**) present the annual SST trends (°C/decade) during the three periods. We used the esd R-package (https://doi.org/10.5281/zenodo.29385) to create the maps in b-f. The maps in a, g-l were generated using Python 3.6 (http://www.python.org) including pyresample 1.19 and cartopy 0.18.

Our initial analyses were based on a suite of global reanalyses and show that the increase in the Arctic SATs over the period 1981–2020 was not spatially uniform. In general, higher positive SAT trends occurred in the marginal seas of the Arctic Ocean with seasonally ice-covered sea. The trends were strongest in the NBS, with an annual warming rate generally between 1.2 °C and 2.0 °C per decade, depending on the reanalysis product (Fig. 1a–d). The maximum annual warming occurred in a zone between FJL and Novaya Zemlya (for location see Fig. 2a) with values ranging from 1.8 °C (Fig. 1b) to 2.5 °C (Fig. 1a) per decade. Here also the largest decline in SIC (Fig. 1e) was observed, with a reduction of up to 16% per decade. Finally, the trends in SST were also pronounced in the Barents area, with the highest trends of 0.5 to 0.6 °C per decade found south of and along the west coast of Svalbard and in the central and south-eastern areas of the Barents Sea (Fig. 1f).

CARRA and ERA5 reanalyses have been used in the further analysis of the Barents study area, and we performed detailed analysis in the following three main regions; *Svalbard, Northern Barents Sea* (NBS) and *Franz Josef Land* (FJL) (Fig. 2a). ERA5 showed higher warming rates in the periods 1991–2020 and 2001–2020 compared to 1981–2020, and the most pronounced warming took place in the eastern NBS and northeast of Svalbard (Fig. 2b–f).

However, CARRA showed notably greater regional SAT trends compared with ERA5. While ERA5 shows an annual warming rate of up to 2.5 °C per decade in both periods, the highest warming rate found for CARRA is 3.1 °C per decade (Fig. 2b–f). The hotspot in ERA5 primarily occurs in a zone between FJL and Novaya Zemlya, but CARRA has in addition a distinct hotspot area between northeastern Svalbard and FJL, especially in the period 2001–2020 with warming rates between 2.5 and 3.0 °C per decade.

#### SAT development from instrumental observations

**Figure 3:**
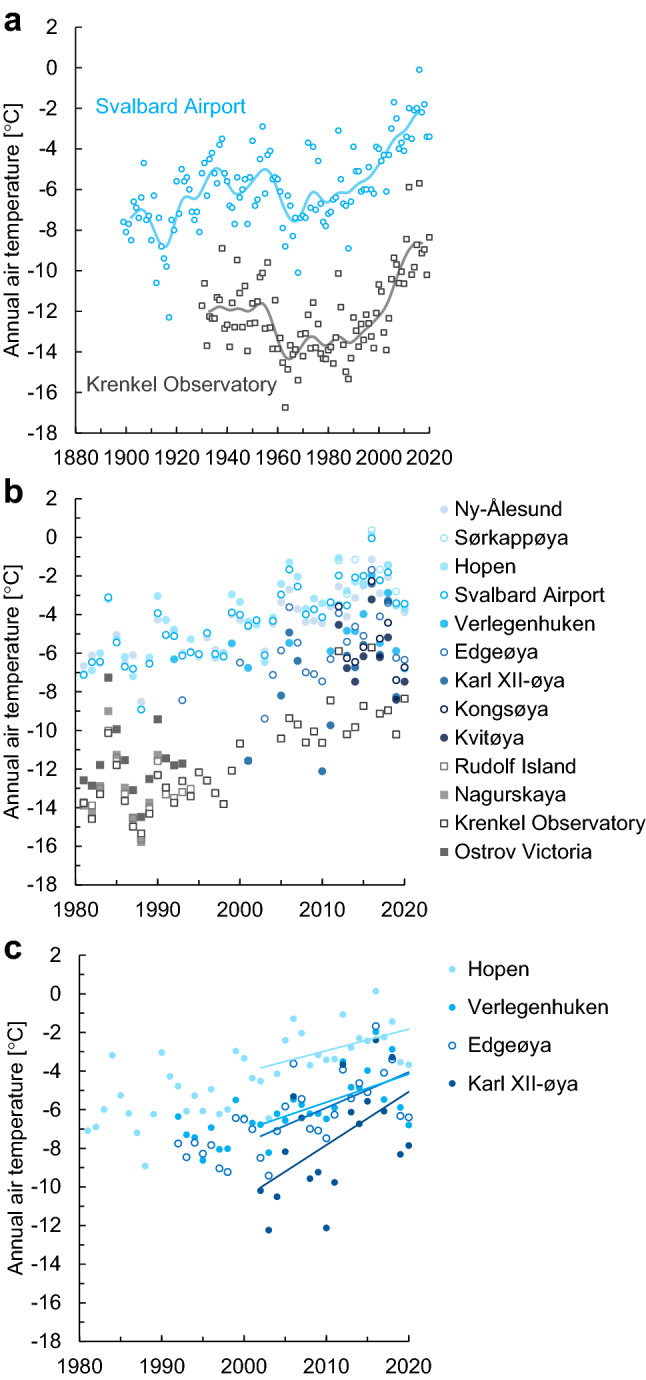
Temperature series from weather stations and observed trends. (**a**) Annual SAT series from the manned stations at Svalbard Airport^24,31^ and Krenkel Observatory^38^. (**b**) Annual SAT 1981–2020 from both old and newly available observations from Norwegian (in blue) and Russian (in grey) weather stations. (**c**) The new SAT series from the automatic weather stations at Verlegenhuken, Edgeøya, and Karl XII-øya compared with the manned station at Hopen. The data in **a** were filtered by a Gaussian filter with a standard deviation of three years, which illustrates variability at a decadal scale. In **c** linear trends for the 2001–2020 period are shown as solid lines. Values for the linear trends and statistical significance can be found in Table 1.

Within the study area (Fig. 2a), two long-term composite series have been established for Svalbard Airport^24^ in the west and the Krenkel Observatory^38^ in the northeast (Fig. 3a). Both highlight the unprecedented high temperatures of the twenty-first century and the recent warming rate that is stronger and longer lasting than during the “early 20th-century warming” (cf. Førland et al.^22^). The annual average temperature for all 13 stations in the study area was compiled for the years they are operated, during 1981–2020 (Fig. 3b). There is an annual temperature difference of more than 10 °C between the coldest stations on FJL and the warmest stations on Svalbard, thus they cover most of the temperature range identified by the reanalyses in the region.

The new instrumental series from northern and eastern Svalbard at Verlegenhuken, Edgeøya and Karl XII-øya (for location see Fig. 2a) were compared with the well-established series from Hopen at south-eastern Svalbard (Fig. 3c). Compared with Hopen, the trend is stronger for all three sites, especially in the recent period 2001–2020. Trend values from the three new series were compared with our four longest time series (Table 1). From 1981 to 2020, our observed station-based data showed an annual warming rate varying between ~ 1.0 °C and 1.6 °C per decade. Record-high warming was observed over the two periods 1991–2020 and 2001–2020, with annual values ranging from ~ 1.1 °C per decade in Ny-Ålesund to 2.7 °C per decade at Karl XII-øya (Table 1 and Fig. 3c). The annual warming was dominated by higher autumn and winter warming but enhanced warming occurred in all seasons (Table 1). In autumn (SON) we noticed an accelerated warming for 1991–2020 and 2001–2020, with up to 4.0 °C per decade for the latter period at Karl XII-øya. In winter (DJF), the highest trends (up to 3.8 °C per decade at Krenkel Observatory) were found for the period 1991–2020. In spring (MAM) the highest trend was observed at Krenkel Observatory, with 2.1 °C per decade during the latest period 2001–2020. All stations showed low or moderate warming rates during summer (JJA), with values ranging from 0 to 0.7 °C per decade. The exception was Karl XII-øya, with a summer warming rate of 1.3 °C per decade during 2001–2020.

**Table 1:**
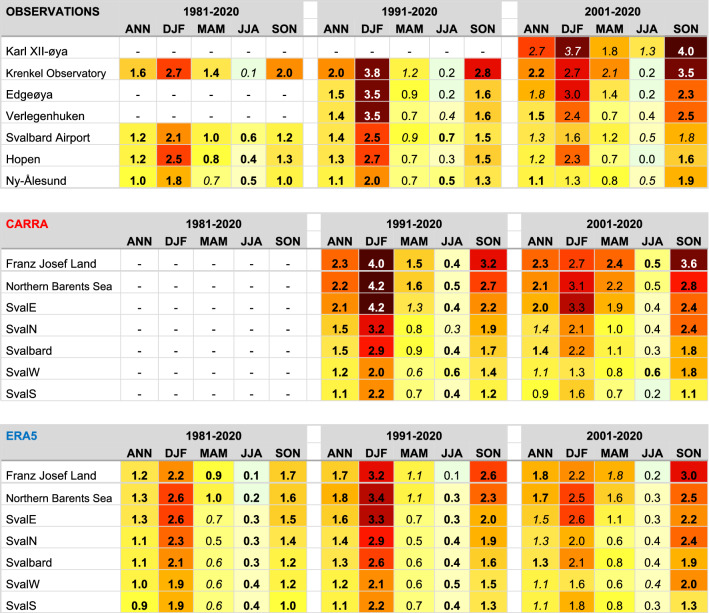
Linear temperature trends (°C/decade) based on instrumental observations and CARRA and ERA5 reanalysis.

Upper panel: Trends are presented for SAT series with complete data (original and interpolated, see Methods) for all or some of the periods 1981–2020, 1991–2020, and 2001–2020, respectively. SAT series from the remaining six stations (with data gaps) are not included here, but are found in Figs. 3 and 6. Middle and lower panel: Regional trends based on CARRA and ERA5, respectively. Dashes indicate not available data for the selected period. Trends that are statistically significant according to a Mann-Kendall test at levels 1% and 5% are marked in bold and *italics*, respectively. Background colours follow the colour scale for SAT in Fig. 2. The text in white is used for better readability against the dark red and brown background colours.

The annual trends were nearly all statistically significant at the 1 or 5% level for the three periods (Table 1). For 1981–2020 and 1991–2020, they were statistically significant at the 1% level. For most stations, seasonal trends were statistically significant at 1% or 5% level for the two longest periods. For 2001–2020, most of the SON trends were significant at the 1% level but for the other seasons either significant at the 5% level or not significant.

#### Comparing observed SAT trends with reanalyses

**Figure 4:**
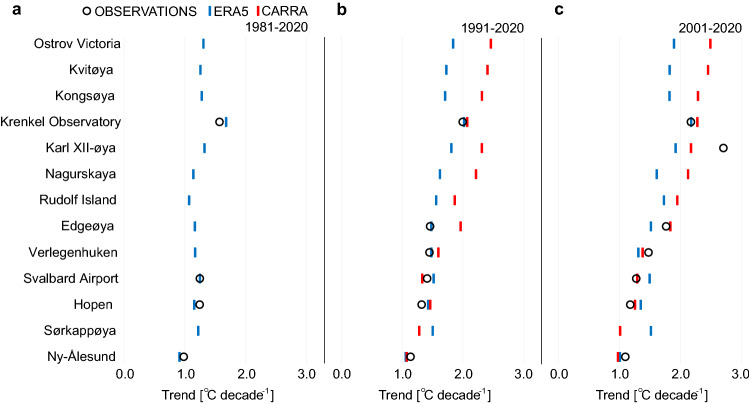
Annual trends in surface air temperature compared with reanalyses. Estimated SAT trends from observations (circles), ERA5 (blue bars) and CARRA (red bars, only in b and c) for the three different time periods: (**a**) 1981–2020, (**b**) 1991–2020 and **c** 2001–2020. The order of stations follows the ranked annual trends computed from CARRA reanalysis for the period 2001–2020. Similar results but for seasonal values can be found in Fig. S6.

The warming seen in the instrumental observations (Table 1) is generally consistent with the spatial and temporal patterns found in the reanalyses. SAT increased in the entire area. The SAT trends were highest in the east and north and lowest in west and south. In the longest period 1981–2020, there was a good agreement between the four observed series and ERA5 (Figs. 4 and S6). For the 1991–2020 period there was also a reasonable agreement between observed trends and both reanalyses. The exception was Edgeøya and partly at Verlegenhuken, where CARRA indicated larger warming than observed (0.5 °C and 0.1 °C higher, respectively). For the period 2001–2020, observations are closer to CARRA than ERA5 at Verlegenhuken, Edgeøya and Karl XII-øya (Figs. 4 and S6). At Karl XII-øya, observed trends are larger than trends from CARRA. For the two latter periods, there is a striking difference between CARRA and ERA5. CARRA generally indicates larger warming rates (of up to 2.6 °C per decade at Ostrov Victoria) than ERA5 for the northern and eastern sites (cf. Figure 2). For the three southwestern stations Hopen, Sørkappøya, and Svalbard Airport, the trends were somewhat larger in ERA5 than in CARRA. Hence, CARRA clearly shows higher spatial variations in the trends than ERA5.

### Evaluation of ERA5 and CARRA reanalyses

The newly established station series from Karl XII-øya, Edgeøya, and Verlegenhuken provide a possibility to assess CARRA and ERA5 reanalyses with new observations that were not available during their production. The results were summarized in terms of biases and Standard Deviations of Error (SDE) in Table 2. It should be noted when comparing reanalysis (grid values) with point observations that some of the differences are due to what they represent and not necessarily errors in the data sets. For CARRA, the biases increase substantially at Karl XII-øya for the period without available observations. However, at Edgeøya and Verlegenhuken the biases are more similar for the two periods. For ERA5, the biases at Karl XII-øya and Edgeøya (NDJFMA only) are substantially higher for the periods where the observations are not available. Furthermore, both reanalyses show higher SDE at all three sites when no observations were available compared to when they were available. However, the availability of observations alone is not enough to ensure that they are assimilated in the reanalyses. For example, adjustments of surface temperatures, based on SAT through the surface assimilation process, are only done for the land part of a grid cell, and in ERA5 only if the land part is larger than 50%^9^. Due to this protocol, local observations of SAT were used at Verlegenhuken and Edgeøya in CARRA, but at Karl XII-øya, which is an ocean point in both reanalyses, the observations were not used in the assimilation process. The same applies to Edgeøya and Verlegenhuken in ERA5 (land part less than 50%). The change in the deviation between the reanalyses and the observations for these sites must therefore be explained otherwise. The mean temperatures, given in Table 2, differ substantially between the periods and support the idea that temporal variations in weather conditions may be the reason for these differences. The SAT in periods without available local observations is on average 6.9 °C, 3.3 °C and 0.9 °C lower for Karl XII-øya, Edgeøya, and Verlegenhuken, respectively. The deviations from the observations in Table 2 for CARRA are consistently similar or lower than the deviations for ERA5 and the results are therefore in good agreement with the general picture on how CARRA adds value to ERA5^39^. Even if both reanalyses show a high correlation with in-situ observations, CARRA shows higher correlations than ERA5, especially for the warmest months May–October (Fig. S5). Obviously, ERA5 produces some of its highest correlations with the observations at Krenkel Observatory and Ny-Ålesund which are the only two SAT observation sites that are assimilated in ERA5 in the region.

**Table 2:** Summary of SAT biases and Standard Deviation of Error (SDE) in the CARRA and ERA5 data sets at three observation sites (Karl XII-øya, Verlegenhuken, and Edgeøya) for the period 1998–2018.

	CARRA				ERA5				Observed SAT	
	Available		Not available		Available		Not available		Available	Not available
	Bias	SDE	Bias	SDE	Bias	SDE	Bias	SDE		
**November–April**										
Karl XII − øya	0.2	0.9 (0.11)	2.9	4.0 (0.37)	0.7	1.9 (0.24)	4.0	4.5 (0.42)	− 10.0	− 16.9
Edgeøya	− 0.3	0.9 (0.12)	0.0	2.9 (0.34)	0.1	1.7 (0.24)	1.2	2.9 (0.34)	− 10.6	− 13.9
Verlegenhuken	0.6	0.7 (0.11)	0.3	1.9 (0.28)	0.8	1.4 (0.21)	0.5	1.9 (0.28)	− 10.5	− 11.4
**May–October**										
Karl XII − øya	0.0	0.5 (0.13)	1.7	2.2 (0.60)	0.4	1.1 (0.27)	2.1	2.0 (0.54)	− 1.4	− 3.2
Edgeøya	0.1	0.6 (0.14)	− 0.1	1.6 (0.36)	− 0.4	1.2 (0.30)	− 0.5	1.7 (0.37)	0.2	0.0
Verlegenhuken	0.3	0.6 (0.14)	0.2	1.2 (0.25)	0.0	1.1 (0.27)	− 0.3	1.5 (0.32)	0.1	− 0.6

Biases (°C) and SDE (°C) are split into different seasons (NDJFMA and MJJASO) and compared to periods where observations are available/not available, for the production of CARRA and ERA5. The values in brackets show SDE (unitless) standardised with the variability (standard deviation) of the observations. The averaged observed SAT (°C) for each period is shown in separate columns.

Additionally, we compared CARRA and ERA5 with observed SAT from ten observation sites, stratified by the nearby sea ice conditions (Table S3). For all sites, the SDE was higher when close ice (SIC > 70%) was present compared to open water (SIC < 10%). The absolute biases were on average only slightly larger for close ice than for open water for both reanalyses, but some locations (e.g. Karl XII-øya) were highly sensitive to the sea ice conditions. Furthermore, the frequency of close or mixed ice (SIC = 10–70%) at Karl XII-øya was higher in the period without available SAT observations (mean SIC = 79%) than in the period with available SAT observations (mean SIC = 50%) and can explain a substantial part of the differences in bias and SDE for Karl XII-øya seen in Table 2. Thus, a pronounced warm bias for close ice conditions was observed. Averaged over all sites, CARRA has a lower absolute bias than ERA5 independent of close ice (0.6 °C) or open water (0.7 °C) conditions.

### Trends in SIC and SST

#### SIC and SST development from remote sensing observations

**Figure 5:**
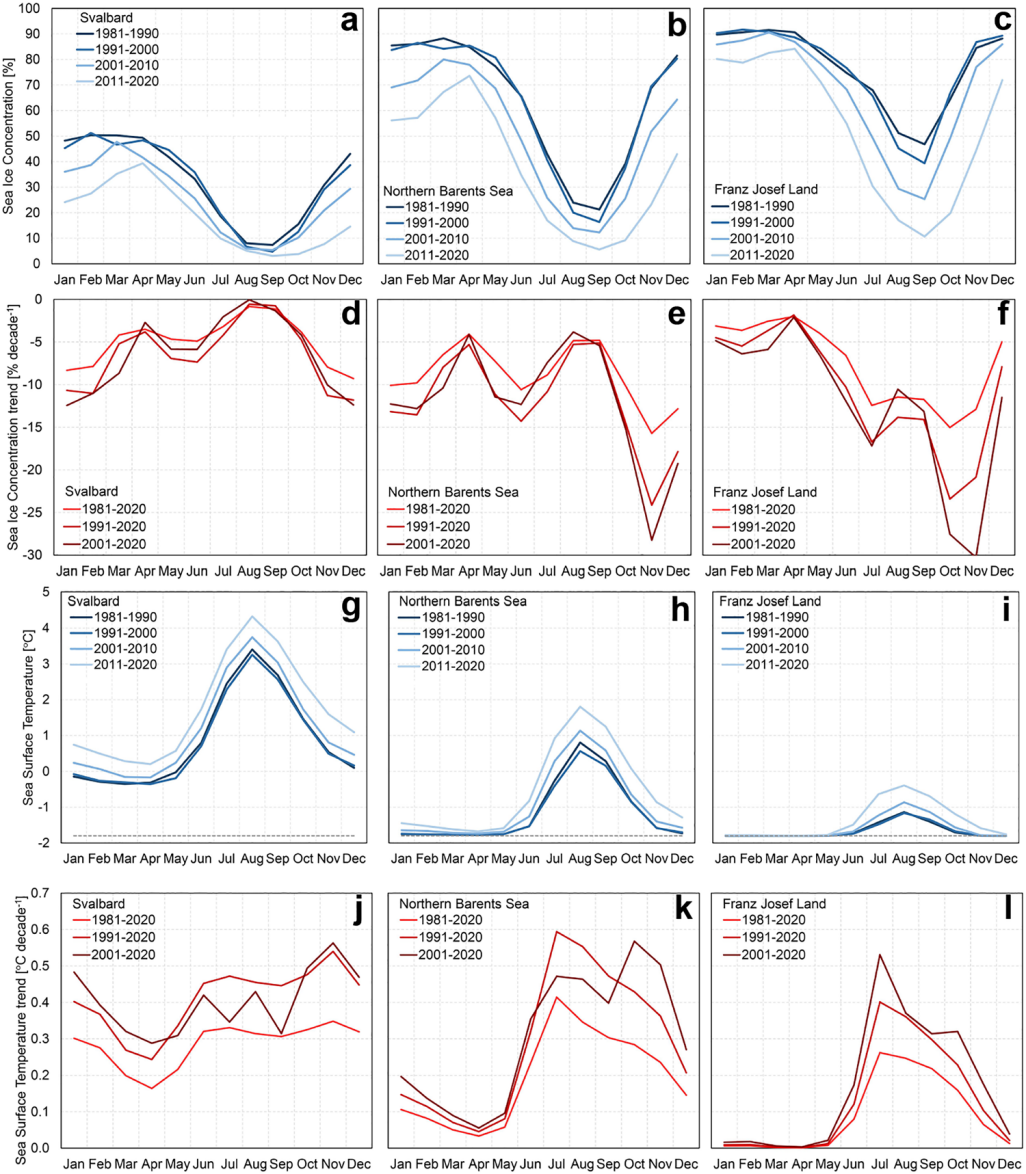
Decadal monthly mean sea-ice concentration and sea surface temperature and trends. Monthly decadal mean SIC and SST for Svalbard (**a**), (**g**), Northern Barents Sea (**b**), (**h**) and Franz Josef Land (**c**), (**i**) (see Fig. 2a). Estimated linear trends for SIC (**d**)–(**f**) and SST (**j**)**–(l**), respectively, for the same areas and for the three study periods.

The sea ice has declined since 1981 in the entire Barents study area (Figs. 2, 5). The strongest decline occurred over the shelf region north of the mean ice edge (Fig. 2) in the NBS with pronounced sea ice reductions in the area between FJL and Novaya Zemlya, and in the area north and east of Svalbard. Similar to the warming pattern, the decline in SIC was more pronounced during the periods 1991–2020 and 2001–2020 than during the entire 40 years period (1981–2020).

The Svalbard region is largely affected by the warm Atlantic Water, making the western part mostly ice-free year-round (Fig. S7) and the whole region is close to ice-free during mid-summer (Fig. 5a). The largest decline has occurred in winter (Fig. 5d) where the mean SIC has decreased from 40 to 50% in the 80 s to 15–25% in the period 2011–2020 (Fig. 5a). The NBS and FJL regions have a generally higher mean SIC throughout the year and with winter SIC (November to May) well above 70% in the 80 s and 90 s (Fig. 5b–c). The NBS SIC has dropped by 5–15% per decade for all seasons, except for November when SIC decreased by more than 23% per decade during the last three decades and more than 27% the recent two decades (Fig. 5e). In FJL, the least decline in sea ice was during winter, however, a strongly reduced ice cover has occurred for the months from June to November with a pronounced and increasingly faster reduction in October–November (Fig. 5f).

The SST trends are overall positive in the Barents study area with the highest warming rates generally found in the third period (2001–2020) along the two branches of the Norwegian Atlantic Current. One of the branches flows along western Svalbard and the other crosses the Barents Sea towards the Arctic Ocean between FJL and Novaya Zemlya (Figs. 2j–l, 5j–l). Of the regions analyzed here, Svalbard west (Fig. 2a) had the highest trends with up to 0.8 °C per decade annually and 1.0 °C per decade in spring (Table 3). Svalbard south, related to the bathymetric trough Storfjordrenna (just south of the station Sørkappøya), also showed an accelerated warming trend (Fig. 2j–l). In the Barents Sea, a very strong warming (up to 0.8 °C per decade) occurred in the southeastern region just outside of the mean ice zone. Furthermore, the southwestern Kara Sea within the mean ice zone experienced a strong warming especially in the last two decades. The weakest SST trends were found for the FJL region ranging from nearly 0 °C per decade in winter and spring to 0.3–0.4 °C per decade in summer and autumn for 2001–2020 (Table 3).

**Table 3:**
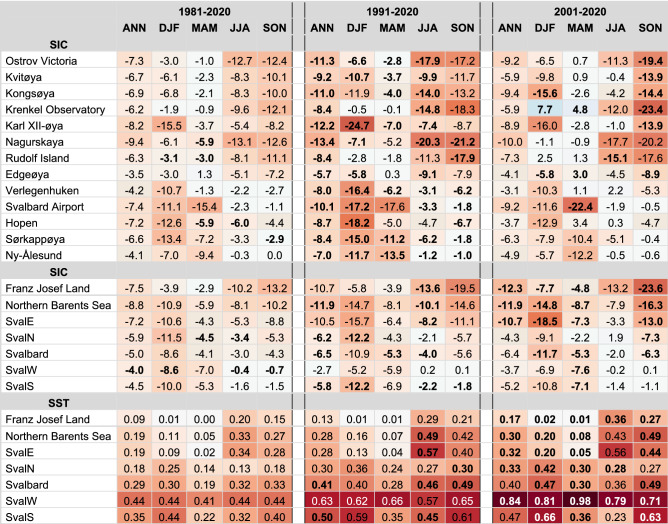
Trends in sea ice concentration and sea surface temperature.

SIC trends (%/decade) estimated from 50 × 50 km boxes around the weather stations (upper part of the table) and trends in SIC (%/decade) and SST (°C/decade) for the regional boxes shown in Fig. 2a (middle and lower part of the table, respectively). Order of station same as in Fig. 4. Background colours follow the colour scale for SIC and SST in Fig. 2. The text in white colours is used for better readability against the dark background colour. The period with the largest trend for each station and region is marked in bold.

#### Observed warming related to sea ice and SST

**Figure 6:**
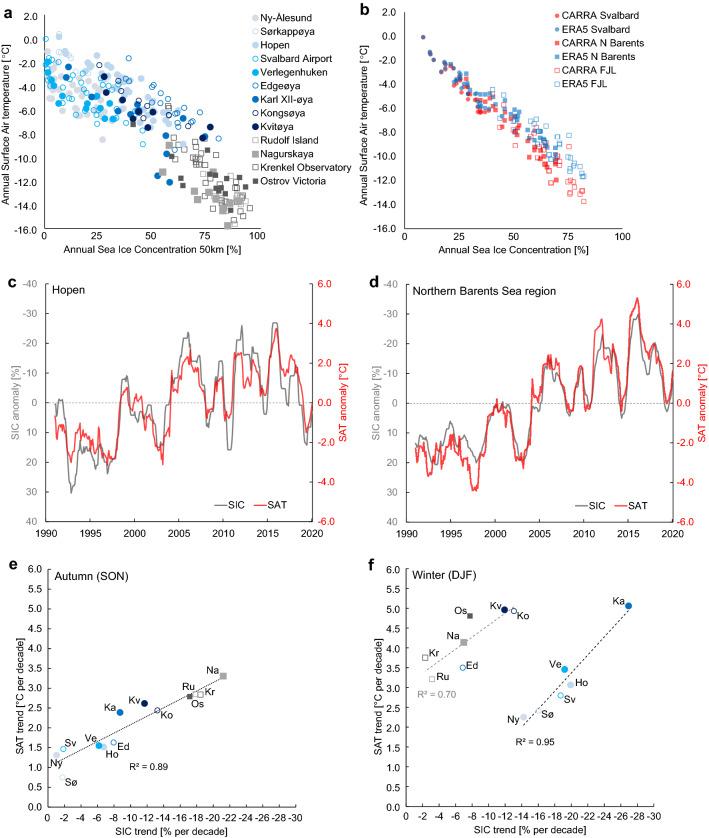
Correlation between sea ice concentration and surface air temperature. (**a**) Scatter plot between the annual SIC (calculated from 50 × 50 km boxes) and the annual SAT observed at the weather stations for 1981–2020. SAT data are original values only (no interpolations). (**b**) Scatter plot between the regional annual mean SIC (OSI SAF data) and SAT (from CARRA and ERA5) calculated from regional boxes (cf. Figure 2a) for the period 1991–2020. **c** 12-month running mean SIC- and SAT-anomalies for Hopen, based on the SIC 50 × 50 km boxes (NIS-dataset) and instrumental observations, respectively. The anomalies were computed with respect to the 1991–2020 mean. (**d**) Same as in c but for the NBS region, based on OSI SAF and CARRA datasets, respectively. (**e**) Scatter plot between 1991–2020 trends in SIC (calculated from 50 × 50 km boxes) and SAT from the weather stations for autumn (SON). (**f**) Same as in **e** but for winter (DJF) with regression lines for stations located in “Svalbard south, west and north” and “Svalbard east and FJL”. In (**e**) and (**f**) CARRA series at station locations is used for gap-filling where SAT data are missing in part of the series for Sørkappøya (Sø), Karl XII-øya (Ka), Kongsøya (Ko), Kvitøya (Kv), Rudolf Island (Ru), Nagurskaya (Na) and Ostrov Viktoria (Os). Stations are shown with symbols and the first two letters of the station name from (**a**).

The spatial pattern of SIC trends resembles very much the SAT trends pattern across the Barents study area (Fig. 2). Figure 6a shows the scatter plot of annual SAT values from the 13 weather stations as a function of the annual SIC in the adjacent sea region (50 × 50 km) extracted from the high-resolution NIS ice charts. The values align well relating low SIC with higher SAT values and vice versa. The FJL stations, typically located further away from the sea ice edge, show a slightly steeper regression between SAT and the ice cover than the Svalbard stations, which more often are surrounded by less dense ice cover.

On a regional scale, the annual SAT derived from both CARRA and ERA5 relate to the annual regional SIC (OSI SAF data) in the same manner as for the station observations (Fig. 6b). However, for the FJL and NBS regions, a stronger disagreement can clearly be seen between the two reanalyses for higher values of SIC (SIC > 50%), with lower SAT values for CARRA compared to ERA5. The lower CARRA temperatures over high SIC for the FJL and NBS regions correspond better with the observed SAT from the associated stations (Fig. 6a).

Both the long-term trend and the interannual variability show high compliance between the 12-month running mean of SIC versus SAT both locally and regionally (Fig. 6c,d). Similar high compliance was found for the two other main regions of Svalbard and FJL. From these time series, the signature warming peaks stand out very clearly for 2006, 2012, 2016 in both SIC and SAT, indicating the absence of a clear temporal lag between the two geophysical properties of sea ice and air temperatures.

Taking into account all 13 station locations, we find that the trends of the annual SAT relate well with the trends of annual SIC (R^2^ ~ 0.5 where R is the Pearson correlation coefficient). It is, however, the autumn and winter seasons that account for most of the high correlation between changes in SAT and SIC. Interactions between local SIC and SAT trends in autumn (SON, Fig. 6e) showed a significant correlation (R^2^ = 0.89) with the strongest warming and sea ice decline occurring for the FJL stations. In winter (DJF, Fig. 6f), a significant correlation was found as well, however, there was a clear regional difference between stations located in Svalbard south-west-north and stations in Svalbard east and FJL. The two latter areas, located in the east, had much lower SIC-trends during winter, than for the Svalbard south-west-north stations despite the as-high SAT trends of similar magnitude (Tables 1, 3). The outcome is shown as two separate regression lines in Fig. 6f for the south-west-north group of stations (R^2^ = 0.95) and the eastern stations (R^2^ = 0.70), respectively.

On a seasonal scale, the SST peaks in August, in close correspondence with the timing of SIC minimum (August–September), see Fig. 5. On the other hand, the SST trend has a remarkable strong peak in July (mainly NBS and FJL) prior to the SST peak, and a second peak in October–November (the third period in NBS and FJL and for all three periods in the Svalbard region) just before the trough of the SIC trend (Fig. 5). The two peaks in the SST trend caused a prolonging period of a warmer sea surface in the summertime. The warming trend in late autumn during 2001–2020 correlated well with the corresponding significant autumn peak seen in the SAT trend (Table 1).

## Discussion

### How do the SAT trends in northern and eastern Svalbard and FJL compare to those in western and southern Svalbard?

Our study showed significant and pronounced warming for the entire Barents area. The annual warming rates accelerated from 1981 to 2020 to 1991–2020 at all stations with sufficiently long series. At the northern and eastern stations, the acceleration continued into the latest period (2001–2020), while the trends dropped more or less off at the stations in the south and the west. These differences in changing trends were mainly connected to conditions in autumn and winter. In winter, the trends during 2001–2020 dropped off after high values during 1991–2020 at all stations. The autumn trends, on the other hand, accelerated everywhere, though less so in the south and the west. While the increased autumn trends in the northern and eastern regions more than compensated for the decreased winter trends, this was not the case at the western and southern stations. A reason for the differences in autumn may be that sea ice has been sparse in autumn in the west and the south during the entire period (Fig. 5a), thus the sea ice trend has been close to zero, while it has been negative elsewhere.

**Figure 7:**
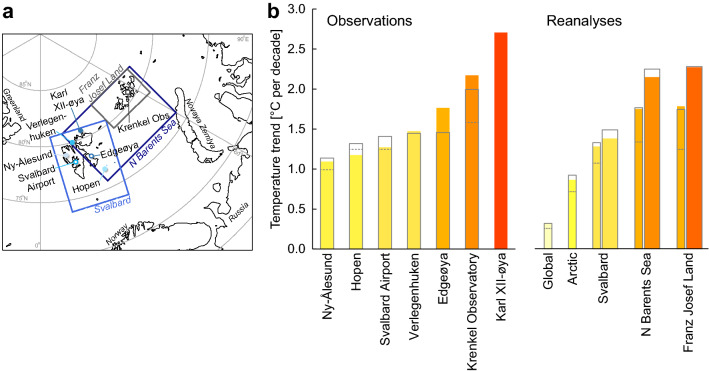
Temperature trends based on instrumental observations and ERA5 and CARRA reanalyses. (**a**) The location of the main stations and main regions in the study. (**b**) Linear trends for annual SAT series and reanalyses for the period 2001–2020. Bar colours follow the colour scale for SAT in Fig. 2. If available, the SAT trends over the 1981–2020 and 1991–2020 periods are additionally shown as dotted and solid grey line bars, respectively. On the right hand side of the bar plots, the ERA5 and CARRA reanalyses are shown as thin and thick bars, respectively. In addition, SAT trends for ERA5 for the Arctic (i.e. north of 65° latitude) and global mean are shown. The map in a was generated using Python version 3.6 (http://www.python.org) including pyresample 1.19.

The accelerated warming up to the latest decade is in agreement with the most recent assessments of instrumental observations in the Arctic^7,8^. Przybylak and Wyszyński^8^ analyzed trends from 1951 to 2015 and showed that the strongest temperature increase in the Arctic in winter was observed over Svalbard, but no stations in north-eastern areas were then available. By including newly available SAT observations from northern and eastern Svalbard and from FJL, we were able to additionally study the regional SAT developments in the NBS. Our main findings are summarised in Fig. 7 and show that the warming in western Svalbard is large, but even larger in northern and eastern Svalbard and in FJL. From 1981 to 2020, we found an annual warming rate varying between 1.0 and 1.6 °C per decade, whereas, over the two periods 1991–2020 and 2001–2020, the annual warming rates ranged from 1.1 to 2.7 °C per decade. These rates are stronger than hitherto known in this region. The increasing temperature rates for the Northern Barents Sea region are exceptional on the Arctic and global scale and correspond to 2 to 2.5 times the Arctic warming averages and 5 to 7 times the global warming averages (Fig. 7).

### How well do reanalyses describe SAT-climatology and SAT trends in the Barents study area, especially for sites and periods without SAT observations available for assimilation?

Our results suggest that both CARRA and ERA5 do a reasonable job in reproducing the observed SAT-climatology and -trends in the high Arctic. However, CARRA seems to represent the SAT more accurately than ERA5, which is in general agreement with Køltzow et al.^39^. However, Køltzow et al.^39^ only verified CARRA and ERA5 with observations that also were available during the production of CARRA. Our results support their findings and indicate that it is also valid for both periods with and without SAT observations available for the production of the reanalyses, and independent of sea ice conditions.

The improved representation of SAT in CARRA originates from a combination of finer resolution, a better description of cold surfaces, and the use of more local observations^37^. In CARRA the biases did not change between periods with and without available local SAT observations for assimilation, i.e. we did not find evidence indicating that observations used periodically had a large impact on trends in CARRA for the few investigated locations here. However, it should be noted that the SDE is reduced in the periods with assimilated SAT observations. For ERA5, a limited amount of SAT observations are assimilated, but these observations seem important as the affected locations are among the sites with the highest correlation between observations and ERA5. For some locations (e.g. Karl XII-øya), the biases are clearly affected when sea ice is present. This is in agreement with other studies showing discrepancies in ERA5 for low SAT and high SIC values^26,29^. Therefore, it seems that changing sea ice conditions alter the bias by adding an “artificial trend contribution”. This may in turn result in reduced temperature trends in the reanalysis, due to a pronounced warm bias under close ice conditions. This situation is more complex along the coast and fjords of Svalbard, as in addition to the treatment of sea ice and the stable boundary layer, the representation of the coastline (ocean/land contrast) contribute to biases in the reanalysis. Hence, the biases show substantial spatial variability and are difficult to generalize. As CARRA has overall smaller biases than ERA5 (Fig. 6b, Table S3), it may explain why the trends are both stronger in CARRA than in ERA5 (Figs. 2 and 4) and apparently more correct.

### How much of the SAT variability is coupled with the SIC and SST variations?

Our results showed that SST increased by up to 0.8 °C per decade in the latest two decades along the western and southern Svalbard, as well as in the southeastern Barents Sea. This is among the highest sea surface warming rates observed in the Northern Hemisphere, and eight times the global mean area-weighted trend in SST^40^. The increase in SST is most likely associated with the larger and warmer Atlantic Water inflow to the study region^12,17,18,20,41^ and the prolonged summer season with high SST in late-autumn may be a crucial preconditioning for the delayed freeze-up that was observed for the NBS and FJL in this study (cf. Timmermans & Labe^42^).

We demonstrated that the SAT increase is strongly linked, both in space and time, to the large reduction of sea ice in the NBS, especially during autumn and winter. This corresponds well with the earlier findings^10,12^, who pointed out that the loss of sea ice, and thereby the loss of the local freshwater contribution, may destabilize the water column and bring up more heat to the surface from the layer of Atlantic Water. This destabilization results in large heat flux from the ocean to the atmosphere due to a relatively warm ocean in winter.

The high correlation between SAT- and SIC-trends in autumn and winter indicated both a local and regional influence that varies among the two respective seasons (Fig. 6e,f). The high correlations seen in autumn suggest a strong local influence of SAT directly related to the SIC trend near the stations, while the regional dependency seen in winter appears to be controlled by the location of stations and the distance to the sea ice edge. The stations located in Svalbard south, west, and north all have low absolute SIC levels (1991–2020 SIC winter mean of 24–53%) and thus are more directly affected locally by open ice and open sea nearby their location.

We found that the most extreme changes in both SAT and SIC occurred over northern Svalbard at Karl XII-øya, with a winter warming rate of 5.1 °C per decade and a SIC-decline of -24.7% per decade, over the period 1991–2020 (Fig. 6f, Table 3). Sea ice cover variability together with water mass dynamics (e.g. colder surface water masses in summer than in winter, cf. Renner et al.^43^) may explain why Karl XII-øya shows different temperature trends and temperature variability compared to the other stations. Compared with Verlegenhuken, Karl XII-øya is a small island located 180 km further northeast and the area between Verlegenhuken and Karl XII-øya is strongly affected by sharp temperature gradients and trends (Fig. 2c) across the marginal ice zone, especially in winter.

In addition, the strongest SST increase was observed in winter for Svalbard west, upstream the West Spitsbergen Current (with respect to the location of Karl XII-øya). The extreme changes found on Karl XII-øya correspond well with the increased heat transport of Atlantic Water by the West Spitsbergen Current, that has a major impact on the reduction of sea ice north of Svalbard in winter^12,18,43^; the excess of heat transport is providing enough energy to keep the area ice-free for longer periods in winter^44^, which in turn has expanded the ice-free region further east in recent years^12,18,45^. This has led to a large oceanic heat loss to the atmosphere in the entire area (cf. Onarheim et al.^18^; Renner et al.^43^; Skagseth et al.^20^) strongly supported by the recently high winter temperature on the Karl XII-øya.

The stations on eastern Svalbard and FJL are dominated by cold Arctic air and cold and fresh surface water, with low SAT (Fig. 3a,b) and high SIC (winter mean SIC more than 76% for 1991–2020). Although the regional SIC-decline for NBS in winter is large (Table 3), the local sea ice conditions near the eastern stations are represented by high SIC and significantly low SIC trends in winter with a lower correlation with SAT than the western stations (Fig. 6f). The large local SAT increase at these stations, therefore, suggests a different warming mechanism than seen for the other stations. One possible explanation is that the high warming trends seen for Svalbard east and FJL stations can be attributed to changes in winter air mass characteristics associated with air advection from areas with lower-than-normal ice concentration and a larger ratio of open water, near the ice edge in the Barents Sea (Fig. S7). This is supported by earlier findings^33^ based on analyses of air mass characteristics performed on large-scale atmospheric circulation types over western Svalbard. One of their main findings was that due to the winter sea ice decline in the NBS, this region apparently has acted as a major remote heat source area for the recent warming in western Svalbard, with cyclonic air advection from east and northeast (Ec and NEc, respectively) being responsible for a major part of the warming.

Following the earlier analysis on cumulative air temperature anomalies within various atmospheric circulation (AC) patterns^33^, we updated their results by including SAT from the Krenkel Observatory (Fig. S8). We found strikingly similar results for the Krenkel Observatory as for the western Svalbard stations, with the largest warming anomalies accounted for by the Ec and NEc circulation types. Since FJL is located further east, an Ec wind pattern over Svalbard west may result in air advection more from the southeast over FJL, depending on the exact location and size of the low-pressure system. This may suggest that the recently observed strong wintertime atmospheric warming in FJL has been driven by an increased heat exchange due to the diminishing sea ice cover east-southeast of FJL, towards Novaya Zemlya and the Kara Sea. This area is the most extreme hot spot area found in the SAT- and SIC trend maps (Fig. 2), corresponding to the area with the largest modeled heat loss in the Barents Sea^20^ and an extreme winter warming during 2000–2016^46^.

Our results (Fig. 6) document a stronger SIC-SAT relation during the 40-year period 1981–2020 than previously known^12,33,35,47^ for the NBS and contribute to additional knowledge on how the presence of sea ice affects the local and regional SAT. Although it remains unclear whether and to what degree the SAT increase is driving the SIC decrease^35^, or vice-versa^12^ we highlight that the recent warming was punctuated by an increasing intensity of abrupt warming events (Fig. 6c,d), with peaks in 2006, 2012 and 2016. These findings are consistent with research showing that the general warming trend of the Atlantic Water upstream of the Barents study area was disrupted by pulse-like events of abrupt warming and cooling, linked to variability in Atlantic water inflow^10,12,20^. The strong SIC-SAT relation suggests that these SAT extremes both contributed to and in part were caused by extremes in the SIC record (cf. Thoman et al.^1^), with far-reaching effects. For instance, the extreme SAT periods strongly affected the terrestrial environment on Svalbard^34,48–50^. Finally, an accelerated warming in both the sea surface and surface air temperature together with withdrawing sea ice causes feedbacks in the Arctic climate system^47,51^ with faster and larger increases in precipitation^52^ and where rain is projected to become the dominant form of precipitation towards the end of the twenty-first century^53^.

## Summary and conclusions

This study has established an extended and consistent high-quality dataset on instrumental surface air temperature (SAT) observed over the period 1981–2020 from the Arctic warming hotspot archipelagos Svalbard and Franz Josef Land in the northern Barents area. We examined the recent warming and its associated variability over the northern Barents area. Further, SAT series from on-site instrumental measurements were compared with the SAT data from the most recent ECMWF reanalysis data set (ERA5) and the recently released regional high-resolution Copernicus Arctic Regional ReAnalysis (CARRA). Furthermore, we related the trend in SAT pattern to variations in Sea Ice Concentration (SIC) and Sea Surface Temperature (SST) based on data from global data sets (EUMETSAT OSI SAF and ESA SST_cci, respectively) and high-resolution ice charts (MET Norway).

We found an unprecedented increase in annual SAT of up to 2.7 °C per decade at Karl XII-øya. The highest warming rates were found in the northern and eastern parts of the Barents area and were up to twice as high than hitherto known in this region from reference station series in the western and southern part. Our results additionally demonstrated that while CARRA and ERA5 reproduced the gross features of the observed trends, CARRA shows more spatial details and larger regional SAT trends. The regional warming rate for the Northern Barents Sea region is exceptional and corresponds to 2 to 2.5 times the Arctic warming averages and 5 to 7 times the global warming averages. Finally, we showed that the warming has been strongly linked, both in space and time, to the large reduction of sea ice and increased SST. Our results also documented a stronger SIC-SAT relation than previously known for the northern Barents Sea with both local and regional relations that varies among seasons.

## Methods

We have focused on three periods, covering 40, 30 and 20 years beginning from 1981, 1991, and 2001, respectively, and all ending in 2020. Starting in 1981, the available records in the first period encompass the pronounced Arctic warming beginning after the 1990s^8,54^, also known as the modern period of Arctic amplification^55^. The 40-year period (1981–2020) covers the modern satellite era including a set of global reanalyses and satellite-retrieved data on sea ice concentration (SIC) and Sea Surface Temperature (SST), whereas, the 30-year period (1991–2020) covers the latest standard reference baseline period from the World Meteorological Organization (WMO) and the Arctic Regional reanalysis CARRA, in addition to two new SAT station series from eastern and northern Svalbard (Edgeøya and Verlegenhuken, see below). The 20-year period (2001–2020) includes the new SAT station series at Karl XII-øya. This is the northernmost (80°39′N) SAT series spanning the most recent period in the Barents region. All three SAT series have been significantly extended in this study (see below). Especially the early part of the three new series was not available during the production of the reanalyses. Moreover, the two latter periods partly covered the years prior to the regime shift on the Arctic SAT time series, with rapid change in mean annual SATs detected in 1995 and 2005^7,8^.

### Instrumental surface air temperature (SAT) data

Russian regular instrumental observations on FJL started in 1929. In this study, four stations were used (Fig. 2a and Table S1). Three of them were closed in the 1990s (Rudolf Island, Ostrov Victoria and Nagurskaya). Only one station (Krenkel Observatory), located in the central part of the archipelago, had been in operation during the whole study period, except for the period 2001–2004. A long-term composite series for Krenkel Observatory has also recently been established^38^ (Fig. 3a).

On the northern and eastern islands of Svalbard, automatic weather stations (AWS) have been in operation since 1991 (Fig. 2a and Table S1). However, during the early years, the data were not stored in MET Norway’s database and there was no quality control at that time. In this work, we have made older data from the 1990s and 2000s available for three stations (Edgeøya, Verlegenhuken and Karl XII-øya) and performed data quality control on three other stations from the 2010s (Kvitøya, Kongsøya and Sørkappøya). The data control was challenging for the oldest part of the data, 1991–2010, in particular before the year 2005, because of the large number and the varying kinds of errors (see Supplementary Information). The most important methods were *limit controls* and so-called *dip tests*. These methods were used stepwise. An additional difficulty was that some observations had slightly wrong time stamps.

The AWS, all in remote areas, are not easily accessible for repair and were often destroyed by polar bears. This resulted in a large number of gaps in the series until a new station setup was developed by the Norwegian Meteorological Institute in 2010 (for details see Supplementary Information).

The missing values were interpolated through linear regression analysis by neighbouring stations and for Verlegenhuken and Karl XII-øya also ice concentration was used (Supplementary Information).

The homogeneity of the series was checked by the recommended homogenization method HOMER^56^. However, the many gaps in the series hampered its use (Supplementary Information). Suspicious values in the final series were extensively discussed and checked (e.g. very low temperatures at Karl XII-øya in November, December, January 2010/2011, and low variability in daily temperatures during summer 2001 and summer 2010–2011). All were found to be reliable.

The significance of temperature trends was studied by the non-parametric Mann–Kendall test, which is a rank test^31,57^.

The new data series from Edgeøya, Verlegenhuken and Karl XII-øya were compared with the long-term, daily SAT series from western Svalbard (Svalbard Airport and Ny-Ålesund) and south-eastern Svalbard (Hopen) (Fig. 3b-c). These series were used as a reference and were earlier scrutinized and homogenized by Førland et al.^22^ and Gjelten et al.^32^. For Svalbard Airport a long-term composite series from 1898 exists^24,31^ (Fig. 3a). All Russian and Norwegian stations are situated close to the sea (see Fig. 2a) at elevations between 5 and 28 m a.s.l. Thus, all stations are influenced by the ocean and sea-ice conditions year-round. Svalbard Airport and Ny-Ålesund are the most “continental” stations, lying in the fjords Isfjorden and Kongsfjorden, respectively.

According to Starkweather et al.^58^, there have been improvements in the quality of Arctic air temperature forecasts and reanalyses since 2005, particularly downstream of automatic weather station (AWS) sites. The series from the AWS stations on the Svalbard north and east used in this study have been upgraded in recent years and are being continued. These data, together with the other Svalbard stations and the Krenkel Observatory on Franz Josef Land, will be important in documenting climate changes as well as central in evaluating and further developing weather models, reanalyses and climate models in this hot-spot area of the Arctic.

### Reanalyses

We used a set of global reanalyses, that have already been widely employed for the Arctic, to evaluate the spatial pattern of Arctic warming (Fig. 1), i.e. NCEP-reanalyses^59^ (the National Centers for Environmental Prediction (NCEP) and the National Center for Atmospheric Research (NCAR), NOAA/ESRL Physical Sciences Division), MERRA-2^60^ (NASA’s Global Modeling and Assimilation Office (GMAO)), JRA55^61^ (the Japan Meteorological Agency (JMA)) and ERA5^9^ (the C3S/European Centre for Medium-Range Weather Forecasts (ECMWF)). No remapping into the same horizontal grid resolution was performed, i.e. the results were mapped onto different grid resolutions.

In further analyses, we used ERA5^9^ and the recently released high-resolution C3S Arctic Regional ReAnalysis (CARRA^37^). The ERA5 dataset covers the whole period 1981–2020, and compared to other global reanalyses ERA5 performs well in the Arctic, with the largest improvements in the wind and temperature fields^28^. CARRA covers the period 1991–2020. ERA5 provides lateral boundary conditions to the CARRA reanalyses.

In our study, CARRA and ERA5 were compared with daily mean SAT observations at three locations for recent periods (mainly after 2010, observations were used in CARRA, but not in ERA5 due to land-fraction below 50%), and for earlier periods (mainly before 2010, observations not available in either CARRA or ERA5) in northern and eastern Svalbard. Further, the representation of SAT over sea ice has earlier been shown to be a weakness in many reanalyses^26,27,29,30^. Therefore, we additionally investigated how the presence of nearby sea ice impacted differences in SAT between observations and the two reanalyses at ten observation sites (Table S3). Both the availability of observations for assimilation in given periods and the presence of sea ice may give periodically changed biases, which ultimately may have had an impact on the SAT trends in the reanalyses.

For most of the periods with missing observations, the differences between CARRA and ERA5 and the interpolated observations were similar to the differences when compared to on-site observations. However, at a few locations and seasons, and in particular at Edgeøya during NDJFMA (mainly January and February 1998), the deviations from the observations in CARRA, and partly ERA5 were different and larger than in other periods. This suggests that there are issues with either the reanalyses or the interpolated observations for this period that need further investigations.

### Sea ice concentration (SIC) and sea surface temperature (SST)

For sea ice concentration (SIC) and sea surface temperature (SST) we used satellite-retrieved data. The global sea ice concentration climate data record, v2.0, consists of daily SIC data from 1979 to the present day and is produced by the EUMETSAT Ocean and Sea Ice Satellite Application Facility (OSI SAF, https://osi-saf.eumetsat.int/). The SIC is based on passive microwave radiometers data and is provided on a 25 × 25 km spatial grid^62^. In order to study the mean ice conditions and trends, monthly and annual averages are computed from the daily values and linear trends calculated over the period of interest (Figs. 1e, 2g–i, 5, 6b–d, Table 3, S7). Regional sea ice time series were computed by averaging the daily SIC over the three predefined main regions (Svalbard, NBS and FJL) and four sub-regions around Svalbard (SvalN, SvalE, SvalS, SvalW, Fig. 2a, Table 3). Decadal trends were generated from these mean SIC time series for the three periods of interest.

Due to the low resolution, these data cannot be used in detailed studies within fjords or close to the coastline. Information from high-resolution navigational ice charts has been used to study the ice conditions closest to the individual SAT land stations and was used to calculate SIC changes and trends (Fig. 6a, Table 3). Ice charts have been routinely produced by the Norwegian Ice Service at MET Norway (NIS, https://cryo.met.no/en/ice-service) since the winter of 1969/70. In the beginning, the charts were based on analogue infrared satellite images, but since the summer of 2007 increasing volumes of high resolution synthetic aperture radar (SAR) satellite data have become available. More details can be found in Hughes and Wagner^63^. The NIS-dataset has been used to calculate daily SIC at a local scale around all the weather stations for the period 1981–2020 (Fig. 5a,c,e,f, Table 3). Time series were produced based on 50 km × 50 km and 100 km × 100 km boxes centered around the stations. Land areas were masked out and only data with zero land fraction in the gridded ice charts were used. Our analyses showed only minor differences between the mean SIC-series obtained from the 2500 and 10,000 km^2^ boxes.

To evaluate the spatial pattern of the SST trends (Figs. 1f, 2j–l, Table 3) we used a gap-free climate data record of global sea surface temperatures that represents a multi-satellite estimate of daily mean SST at 20 cm depth with a feature resolution of about 20 km (0.05 degree on a regular latitude–longitude grid) derived from satellite infrared observations^64^. The dataset has been produced as part of the European Space Agency (ESA) Climate Change Initiative Sea Surface Temperature project (ESA SST_cci, v2.1) and covers the period 1981–2016. The remaining years, 2017–2020, are taken from the interim climate data record v2.0 extension which is generated under Copernicus Climate Change Service (C3S) and is available from the C3S climate data store. Temporal and spatial averages are computed from SST in a similar manner as for the OSI SAF SIC. Note, that in these average calculations, SST values below sea ice—fixed at − 1.8 °C—are included.

## Supplementary Information

Supplementary Information.

## Data Availability

The datasets generated and/or analysed during the current study are available in the following repositories: All SAT data based on observations from norwegian weather stations can be downloaded from MET Norway's archive of historical weather and climate data: https://seklima.met.no/ and https://frost.met.no. Surface Air Temperature observations from the Russian weather stations can be downloaded from the Russian Scientific Research Hydrometeorological Institute (www.meteo.ru), AARI archives of historical weather and climate data (www.aari.ru) and Russian patent archive (https://www1.fips.ru/publication-web/publications). The 2 m air temperature derived from reanalyses data such as ERAINT, ERA5, NCEP, and CARRA data can be downloaded from the COPERNICUS climate data store at https://cds.climate.copernicus.eu The Japanese 55-year Reanalysis (JRA-55) can be downloaded from the research data archive—Computational and information system lab at https://rda.ucar.edu/datasets/ds628.0/. The Modern-Era Retrospective analysis for Research and Applications, Version 2 (MERRA2) can be downloaded from the Goddard Earth Sciences data and information Service center at https://disc.gsfc.nasa.gov/datasets?project=MERRA-2. EUMETSAT Ocean and Sea Ice Satellite Application Facility, global sea ice concentration climate data record 1979–2015 (v2.0, 2017), OSI-450, https://doi.org/10.15770/EUM_SAF_OSI_0008. Data can be extracted from the OSI SAF FTP server ftp://osisaf.met.no/reprocessed/ice/conc/v2p0. EUMETSAT Ocean and Sea Ice Satellite Application Facility, Global sea ice concentration interim climate data record 2016-onwards (v2.0, 2017), OSI-430-b. Data can be extracted from the OSI SAF FTP server ftp://osisaf.met.no/reprocessed/ice/conc-cont-reproc/v2p0/. High-resolution navigational ice charts produced by the Norwegian Ice Service at MET Norway (NIS) can be downloaded at https://cryo.met.no/en/ice-service. The dataset citation for the CCI L4 CDR v2.1 is: Good, S.A.; Embury, O.; Bulgin, C.E.; Mittaz, J. (2019): ESA Sea Surface Temperature Climate Change Initiative (SST_cci): Level 4 Analysis Climate Data Record, version 2.1. Centre for Environmental Data Analysis, 22 August 2019. Doi: https://doi.org/10.5285/62c0f97b1eac4e0197a674870afe1ee6. https://doi.org/10.5285/62c0f97b1eac4e0197a674870afe1ee6. The SST CDR v2.1 is here supplemented by the interim CDR (ICDR) v2.0 extension which is generated under Copernicus Climate Change Service (C3S) and data is extracted from the C3S climate data store https://cds.climate.copernicus.eu/cdsapp#!/dataset/satellite-sea-surface-temperature?tab=overview.

## Code availability

Information about the codes can be obtained from the corresponding author upon request.
