# Supplementary material for: Exceptional warming over the Barents area
Source: Sci Rep. 2022 Jun 15;12:9371. doi: 10.1038/s41598-022-13568-5 (PMC9200822; doi:10.1038/s41598-022-13568-5)
Supplement: Supplementary file 1 — Supplementary Information. [file 41598_2022_13568_MOESM1_ESM.pdf]

Exceptional warming over the Barents Area

Ketil Isaksen[1], Øyvind Nordli[1], Boris Ivanov[2,3], Morten A.Ø. Køltzow[1], Signe Aaboe[1], Herdis M. Gjelten[1], Abdelkader Mezghani[1], Steinar Eastwood[1], Eirik Førland[1], Rasmus E. Benestad[1], Inger Hanssen-Bauer[1], Ragnar Brækkan[1], Pavel Sviashchennikov[3,2], Valery Demin[4,2], Anastasiia Revina[2] & Tatiana Karandasheva[2]

1 Norwegian Meteorological Institute, Oslo, 0313, Norway

2 Arctic and Antarctic Research Institute, St. Petersburg, 199397, Russia

3 Saint-Petersburg State University, St. Petersburg, 199034, Russia

4 Polar Geophysical Institute, Apatity, 184209, Russia

Supplementary Information

Location of the weather stations and regions used in this study

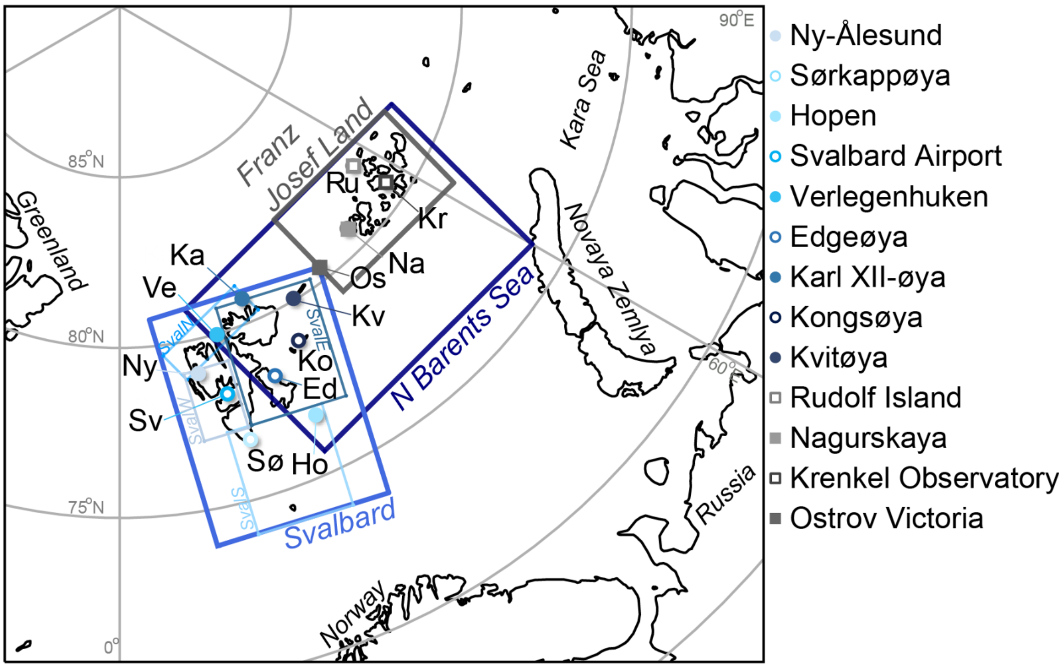

Supplementary Fig. 1 Barents study area and the locations of the regions (boxes) and 13 weather stations. The main regions Svalbard, Northern Barents Sea (NBS), and Franz Josef Land (FJL) are shown with thick line boxes. The sub-Svalbard regions, Svalbard North, East, South and West, are shown as thin line boxes. The weather stations are shown with symbols (grey for Russian stations and blue for Norwegian stations) and the first two letters of the station name from the stations list to the right of the map. The map was generated using Python version 3.6 (http://www.python.org) including pyresample 1.19.

The list below gives the corner coordinates (lon,lat) for each region (box) where 1. is the upper-left corner, 2. the upper-right, 3. the lower-right and 4. the lower-left corner. The map projection used is EASE-Grid 2.0.

Main regions

Svalbard

1 (5.6735496521,80.9780426025), 2 (38.9953727722,80.4770431519),

3 (29.7370910645,73.6406478882), 4 (10.5990095139,73.9273376465).

Northern Barents Sea (NBS)

1 (12.2866716385,80.8999481201), 2 (69.9677658081,81.5423126221),

3 (24.7906703949,75.6823577881), 4 (59.9505500793,76.0828857422).

Franz Josef Land (FJL)

1 (32.789478302,82.0394287109), 2 (67.4433746338,81.5903320312),

3 (35.6029319763,79.6626358032), 4 (62.5267219543,79.3127822876).

Sub-Svalbard regions

SvalNorth

1 (7.26237440109,79.6830291748), 2 (22.2060852051,81.1288757324),

3 (24.3351078033,80.2541046143), 4 (10.222949028,78.9213638306).

SvalEeast

1 (17.829536438,80.6988143921), 2 (34.6972618103,80.2534942627),

3 (30.0747432709,76.8316345215), 4 (17.6040668488,77.157699585).

SvalSouth

1 (14.2927427292,77.0997924805), 2 (26.8424682617,76.9294281006),

3 (25.0042133331,73.8733596802), 4 (14.8049468994,74.0112686157).

SvalWest

1 (10.0361347198,79.0524902344), 2 (16.8814201355,79.1302719116),

3 (16.8996620178,77.1135559082), 4 (11.0995206833,77.0478744507).

Metadata for weather stations used in this study

Supplementary Table 1 Metadata for weather stations used in this study. Station name with the WMO identifier ("index number"), H_s = station altitude, Screen = radiation protection for temperature, H_t = height above the ground for the temperature sensor, system = measuring and/or data transmitting system used. The data periods of surface air temperature (SAT) include only those of accepted quality.

Station (Russian/Norwegian official name, WMO-ID)	Periods (SAT)	System	H_s(m)	Screen	H_t(m)
**Gråhuken**	1991.07-1995.12	CMI/Jotron	10	MET*	4.2
**Verlegenhuken** (01002)	1997.08-2010.08	CMI/Jotron	8	MET*	4.2
	2010.08-present	Setup of 2010	8	MET	4.2
**Edgeøya** (01006)	1992.09-1995.12	CMI/Jotron	14	MET*	4.2
	1997.08-2010.08	Aanderaa	14	MET*	4.2
	2010.08-present	Setup of 2010	14	MET	4.2
**Ny-Ålesund** (01007)	1974.08-present	Manned/AWS	8	MET (MI-46)	2
**Svalbard Airport** (Svalbard lufthavn, 01008)	1975.08-present	Manned/AWS	28	MET (MI-33,MI-74)	2
**Karl XII-øya** (01009)	2000.08-2010.08	Aanderaa	2	Aanderaa	5
	2010.08-present	Setup of 2010	5	MET	4.2
**Kvitøya** (01011)	2010.08-present	Setup of 2010	10	MET	4.2
**Kongsøya** (01016)	2010.09-present	Setup of 2010	20	MET	4
**Sørkappøya** (01020)	2010.09-present	Setup of 2010	10	MET	3.7
**Hopen** (01062)	1945.11-present	Manned	6	MET	2
**Ostrov Viktoria** (20026)	1959.11-1994.03	Manned	15	Stevenson screen	2
**Nagurskaya** (20034)	1952.06-1991-07	Manned	18	Stevenson screen	2
**Krenkel Observatory** (Observatoriya Krenkelya, 20046)	1957.11-present	Manned	21	Stevenson screen	2
**Rudolf Island** (Ostrov Rudolfa, 20049)	1932.10-1995.04	Manned	51	Stevenson screen	2

*A screen for humidity sensors of type Lambrechts, but modified to include both temperature and humidity sensors.

Quality control of Surface Air Temperature (SAT) data

Russian stations

The Russian weather stations included in this analysis are Krenkel Observatory, Nagurskaya, Rudolf Island and Ostrov Victoria. The temperature data from the stations have undergone both manual and automatic quality controls in several stages. The data were initially manually controlled at the weather station by the observers and have later undergone several rounds of manual and automatic quality control including consistency checks and outlier tests.

Tests to identify large errors and suspicious observations in the temperature series included logical tests using differences between maximum, minimum and mean temperature. To identify outliers, Grubbs' criterion was used where values exceeding ±2.5 standard deviation from the monthly mean were marked and examined. A modified Tietjen-Moore test[1], was sometimes used to test outliers. All

suspicious values were examined by experts at AARI (Arctic and Antarctic Research Institute), RIHMI-WDC (All-Russia Research Institute of Hydrometeorological Information - World Data Center) or SPSU (Saint Petersburg State University) who made the final decision on whether to keep or reject the value.

The temperature series were also compared to series from neighboring stations to identify possible systematic errors giving shifts in the data series. The homogenized temperature series from Krenkel Observatory also includes data from the weather station Bukhta Thikaya and has been carefully scrutinized as described by Ivanov et al.[2].

Norwegian stations

Svalbard Airport, Ny-Ålesund and Hopen are weather stations intended for forecasting and climate analysis and the data from these stations undergo extensive quality control (QC) when being stored in MET Norway's database. Quality control has been performed mostly manually until 2005 when an automatic QC routine was put into use that includes several consistency tests such as step tests and threshold tests, in addition to manual inspection of values flagged as suspicious by the system. There have been several changes in instrumentation and location at all three stations leading to breaks in the homogeneity of the series. More details on quality control, station changes, and homogeneity can be found in Førland et al.[3], Nordli et al.[4-5], Gjelten et al.[6], and Hanssen-Bauer et al.[7].

During a time span of nearly thirty years automatic weather stations (AWS) have been in operation on the northern and eastern islands of Svalbard. The instruments and station infrastructure have varied much during those years (**Supplementary Table 1**). During the early years the data were not stored in MET Norway's database, and there was no quality control. There were also problems with the regularity of the data, in particular many stations were destroyed by polar bears. In 1996, no data of accepted quality reached MET Norway. However, in 2010 a new setup of stations was developed, which improved data quality and significantly reduced the number of missing data. Hence, almost all our work on data control for this study was related to data before the autumn of 2010.

Until 2010 the satellite system ARGOS was used for data transmission. It sent messages every 200 seconds. The satellites were used as a "mirror" so when they saw both a station and the MET Norway satellite antenna, the transfer of data took place. The number of satellites varied from 1-2 to optimally 5 at the end of the period. Parts of the day might be without data if using only 1-2 satellites, whereas with 5 satellites the whole day was sufficiently covered.

The aim of the stations was first and foremost to include their data in weather models for improving weather predictions for these remote areas. Therefore synops were generated from the raw data, but the data was not intended for climatology and was not even stored in the database of the institute. This changed in 2003 when Edgeøya station started and in 2007 the first monthly mean temperatures were presented in the database (**Supplementary Fig. 2**). But still the quality was poor, so an extensive quality control program was necessary for obtaining sufficient quality. In this study, the following steps were taken.

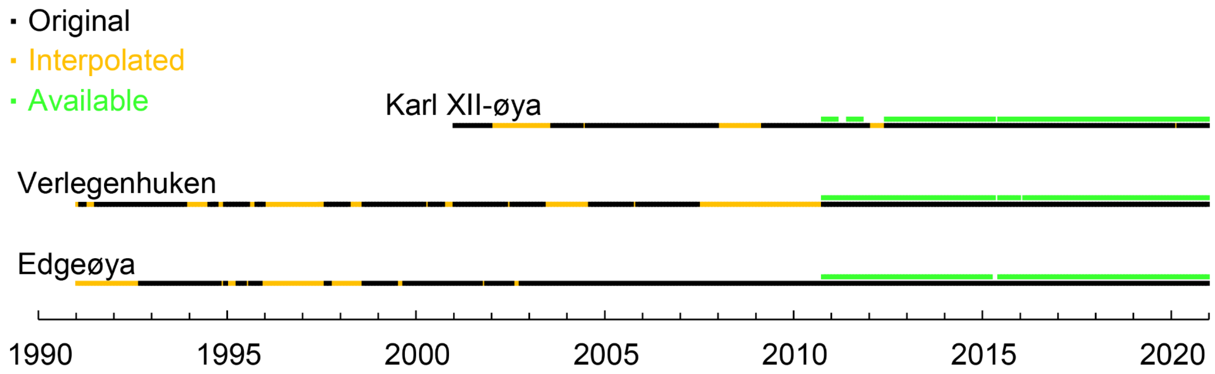

Supplementary Fig. 2 Data coverage for the stations Karl XII-øya, Verlegenhuken and Edgeøya. Periods with original surface air temperature measurements are marked in black. Yellow lines show periods with interpolated values. Green shows periods where data is stored and available in MET Norway's database until this study.

Data testing procedure

Step 1. There were many observations with the same time-stamp without containing identical observations. The most reliable data elements were temperature, T, and pressure, P. If those weather elements were equal, one of the observations was deleted.

Step 2. Some evening observations were dated one day too late. They were corrected mainly by comparing the extreme temperatures. Unfortunately, this was not a minor problem. For example, at Verlegenhuken during the period 1997.08-2005.01, as many as 108 evening observations were dated wrong.

Step 3. Totally unrealistic values of weather elements were deleted after being processed by a limit control.

Step 4. For further testing a dip test was introduced for temperature and pressure. See an example with temperature in equation (1).

$$(T_{-1} - T_0)(T_0 - T_1) < D \qquad (1)$$

T_0 is the temperature under testing, where T_{-1} is the temperature one-time step before and T_1 is the one-time step after T_0. D is a constant which determines how strong the test should be. If the temperature is increasing or decreasing, the product will be positive. If the product is negative then T_0 is either larger or smaller than both of its neighbours, T_{-1} and T_1. If the product is smaller than D, T_0 should be discarded. For example, if D = -400 °C^2 the dip in the measured temperature must be larger than 20 °C for being discarded, so D = -400 °C^2 is not a strong test if the resolution is three hours.

The dip test was used on data having passed step 3. Observations could still have the same time-stamp, but only if T or P were different. Those double data caused problems for the dip test. This was solved by using very weak tests in the beginning and by deleting obvious cases. Stepwise the test was strengthened and for each step, doublets were deleted.

Step 5. When the number of doublets were reduced to a minimum, the rest was subject to manual control. After this step all doublets were removed.

Step 6. With no doublets in the data set, the dip test was further strengthened. The criteria was -100 $°C^2$ for temperature and -100 hPa^2 for pressure.

Step 7. A combination of a limit test and dip test was performed to identify quite a small number of observations, which were subject to manual control, taking into account the weather situation.

Step 8. Logical tests were performed for extreme temperatures, T_{max} or T_{min}. If the test was violated by more than 1°C, the extreme temperature was deleted. In the case of violation by less than 1°C the extreme was adjusted to the highest (lowest) temperature at fixed hours.

Step 9. Relative humidity, U < 20 % or U > 102% was deleted. For 101 % < U < 103 % the value was adjusted to 100 %.

Step 10. Wind force > 40 m/s was deleted. Dip test values less than -400 m^2/s^2 were also deleted.

Calculation of daily mean temperature

The WMO-definition of daily mean temperature, T_m, is based on 24 hourly observations evenly distributed throughout the day, see equation (2).

$$T_m = \frac{1}{24}\sum_{i=1}^{24} T_i \qquad (2)$$

where T_i is the temperature at the *i-th* hour of the day.

From the start of the series to the autumn of 2010 daily mean temperatures were calculated by use of the so-called synops, i.e. observations at the hours 00, 03, 06, 09, 12, 15, 18, 21 UTC, a total of 8 evenly distributed observations throughout the day. For data later than autumn 2010 we followed the WMO standard of 24 daily observations. The RMSE (Root Mean Square Error) in the daily means of 8 observations compared with 24 hours was 0.2°C. We found that using only 8 observations did not lead to biased daily means so no corrections were applied when all synops in a day were available.

However, missing data was very frequent in the early age of the automatic stations. The observations in the evenings were often missing so bias corrections were necessary. An estimate of the daily mean temperature, T_{dm}, with n observations ($n < 24$) using bias corrections is then given by equation (3).

$$T_{dm} = \frac{1}{n}\sum_{i=1}^{n} (T_i + d_{mi}) \qquad (3)$$

where d_{mi} is the bias correction at hour i.

The magnitude of d_{mi} was estimated for all stations by the use of the daily 24 hourly observations (**Supplementary Fig. 3**). The mean bias at hour i, d_{mi} varies with the short wave radiation, being largest in June and smallest in December. In June d_{mi} is in the interval [-0.5, 0.5] °C at Edgeøya and somewhat smaller for the other stations. In December the d_{mi} can be neglected.

During the early stages of AWS at Svalbard synops were often missing, so the daily mean temperatures were often calculated with less than 8 observations during the day. This leads to anomalies compared to the “true” means of 24 observations. There are some seasonal differences of the anomalies, they are largest during winter and smallest during summer. The RMSE compared to 24 observations is about 1 °C if only 1 observation in the day is available, and about 0.5 °C if 4 observations are available (**Supplementary Fig. 4**).

From autumn 2010 the observations from the AWS on Svalbard are included in the database of MET Norway. Occasionally missing observations were interpolated by model data. These were not bias-

corrected in the first years, so using them for the estimation of trends in the data series could be risky. Therefore, we have calculated bias-corrected daily means for the use in this article. However, since 2013 the model data used for interpolations are bias corrected. These we have accepted without any change.

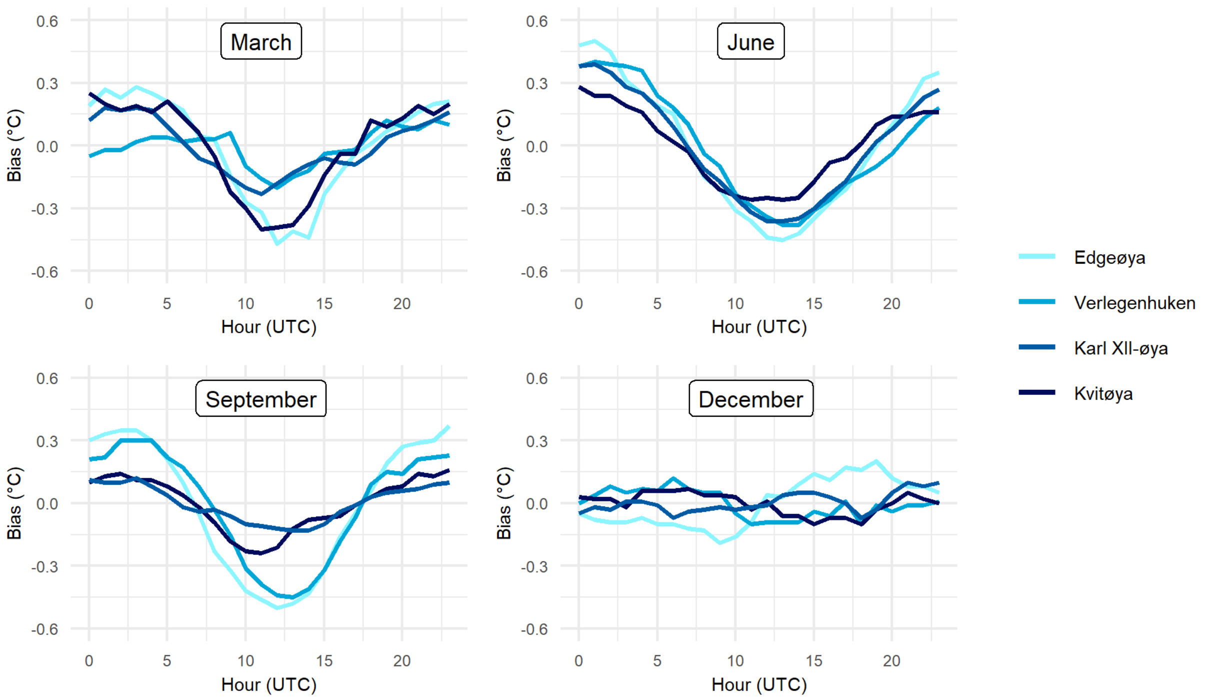

Supplementary Fig. 3 Bias correction for Edgeøya, Verlegenhuken, Karl XII-øya, and Kvitøya. The bias correction for each hour in the day compared to the daily mean value is shown for four selected months (March, June, September and December).

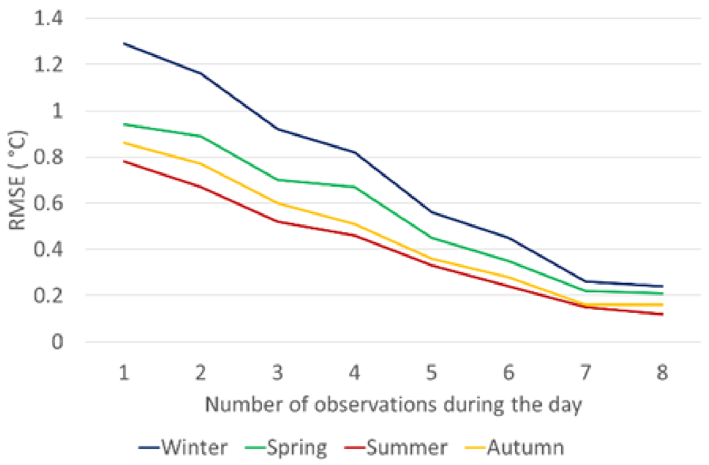

Supplementary Fig. 4 RMSE for the anomaly between "daily means" calculated from available synops during the day compared with true daily means calculated by 24 hourly observations.

Homogeneity testing and further quality control

As there are many known changes at the stations such as relocations and change in equipment, a homogeneity test was performed on the dataset as an additional step in the quality control. This proved to be challenging since the dataset consisted of a few stations with relatively short time series with many gaps. In addition, there are generally few stations at Svalbard and the distances between them are large. This means that the network of reference series used in the homogeneity analysis would be small and have relatively low correlation coefficients. Even so, the homogeneity testing was performed to get an indication of the quality of the time series, possible homogeneity breaks and to identify suspicious values.

The homogeneity testing was performed using the software HOMER[8] with monthly temperature means calculated from the interpolated version of the dataset for Edgeøya, Verlegenhuken and Karl XII-øya. Temperature series from Ny-Ålesund, Svalbard Airport and Hopen were used as additional reference series. The series from Krenkel Observatory was included in a test run but correlations with the series from Svalbard were low (0.78 and lower). Thus, the series was not included in the final runs.

The series from Verlegenhuken was merged with the series from Gråhuken. However, the Gråhuken series had so many missing values that it was not possible to adjust the homogeneity break connected to the merging when using HOMER. This merging was however adjusted for in the interpolation of daily values, see next section.

Verlegenhuken, Edgeøya and Karl XII-øya were all equipped with the new AWS setup in August 2010. Such a change might lead to a homogeneity break in the series, but since the change happened at the same time at all the stations the results from the analysis were ambiguous.

In the end, none of the series were adjusted for homogeneity breaks at this step in the analysis because of gaps in the series, sparse station network, possible breaks being too close to the end of the series, and change of equipment at the same time at the stations. Differences in sea ice conditions also influence the results and may present false homogeneity breaks. This was especially true for Karl XII-øya which is a small island located further northeast and with different ice conditions than the other stations in some periods. Sea ice cover variability together with water mass dynamics (e.g. colder surface water masses in summer than in winter, cf. Renner et al.[9].) may explain why Karl XII-øya shows different temperature patterns in some periods compared to the other stations (e.g. very low temperatures in November, December, January 2010/2011, and low variability in daily temperatures during summer 2001 and summer 2010-2011). The station at Karl XII-øya has also been visited during regular service and inspections (every year or second year) and no significant deviations during on-site calibrations routines or during replacement of air temperature sensors were found. The Norwegian Meteorological Institute has long experience with these sensors and they are found to be stable and reliable.

Interpolation of missing daily mean temperature

It was an extreme challenge to run the remote stations in eastern Svalbard steadily through the polar winter without any possibility to do maintenance work. Not only technical problems caused data loss, but also an even greater problem was damages caused by polar bears. However, after the introduction of a new setup for the construction of Arctic AWS in 2010, the series are almost without gaps.

The two oldest AWS on eastern Svalbard are Gråhuken, starting in July 1991, and Edgeøya – Kapp Heuglin, starting in September 1992 (**Supplementary Table 1**). In August 1997, Gråhuken was closed and replaced by a new station at Verlegenhuken 45 km NE of Gråhuken (**Supplementary Fig. 1**). We wanted to analyse the temperature climate over the thirty-year standard normal period, 1991-2020. Therefore, we linked the two series together into a composite series, hereafter named Verlegenhuken.

The gaps in the Verlegenhuken and Edgeøya series amounted to 28 % and 17 % respectively. They were filled by interpolations from regression analysis (Eqn. 4), using neighbouring stations as predictors. Ice cover around the stations was used as an additional predictor if it improved the interpolations. The validation was based on the variance accounted for by the regression and the RMSE (**Supplementary Table 2**). The interpolations were regressed independently for each month.

Interpolation of missing data, T_{int}, in the AWS temperature series was performed by the use of equation 4:

$$T_{int} = a + bT_2 + cI_1 \qquad (4)$$

where a, b and c are constants and T_2 is the temperature at a neighbouring station and I_1 is the ice cover around the site of the missing data. The constants are coefficients calculated by regression analysis (Wilks 1995) with T_2 and I_1 as predictors. Daily values were used for both temperature and ice cover. The ice cover data were not solely daily, but variations were so slow that missing values could easily be assessed with negligible uncertainty by use of the nearest values in the time series. Each month in the year was treated separately. Therefore, the constants differed from month to month.

The neighbouring stations of Edgeøya are Svalbard Airport (160 km W) and Hopen (200 km SSE). These time series were a priory chosen as predictors in linear regressions for filling the gaps of the Edgeøya series. The two regressions obtained nearly the same skill for most of the months except for the summer months JJA. For those months, Hopen was the better of the two. A two-predictor approach was also tried by using the ice cover around Edgeøya as a predictor together with the temperature at the neighbouring stations. The ice cover was defined as the percent of ice within squares of 50 km^2 and 100 km^2. However, none of them improved the regression so the approach with Hopen as the only predictor was chosen **(Supplementary Table 2).**

Supplementary Table 2 Uncertainty of the interpolations of daily mean temperature in the series from Edgeøya, Verlegenhuken and Karl XII-øya. SSR/SST is the regression sum of squares divided by the total sum of squares (often named the variance accounted for by the regression) whereas RMSE is the Root Mean Square Error.

	Jan	Feb	Mar	Apr	May	Jun	Jul	Aug	Sep	Oct	Nov	Dec
Edgeøya interpolated by Hopen												
**SSR/SST (%)**	81	70	74	79	77	51	34	42	76	81	83	80
**RMSE**	4.1	3.0	4.1	3.0	1.9	1.4	2.0	1.7	1.2	1.9	2.4	3.3
Verlegenhuken interpolated by Ny-Ålesund and ice cover 100 km^2 around Verlegenhuken												
**SSR/SST (%)**	87	83	87	84	76	52	37	44	76	83	86	79
**RMSE**	2.2	3.1	2.6	2.5	2.0	1.4	1.8	1.7	1.4	1.7	2.1	2.6
**Adjustments**				-2.0	-1.7	-2.0	-1.9	-2.3				
Karl XII-øya interpolated by Verlegenhuken and ice cover 100 km^2 around Karl XII-øya												
**SSR/SST (%)**	85	84	85	85	78	34	20	38	61	80	82	80
**RMSE**	4.5	3.5	3.8	3.1	2.0	1.6	1.9	2.1	2.0	2.2	4.7	4.7
Karl XII-øya interpolated by Ny-Ålesund and ice cover 100 km^2 around Karl XII-øya												
**SSR/SST (%)**	80	81	72	73	61	25	15	37	50	63	81	72
**RMSE**	4.5	3.9	4.9	4.2	2.6	1.6	1.8	2.0	2.2	3.1	3.7	5.0

Ny-Ålesund was chosen as a predictor for the interpolations of the gaps in the Verlegenhuken series. It is the nearest station to Verlegenhuken, situated 150 km SW. Testing revealed that it was possible to improve the interpolations during the winter months DJFM by using ice cover over 100 km^2 around Verlegenhuken as an additional predictor. For simplicity, the two-predictor regression was adopted for all months (**Supplementary Table 2**).

Karl XII Island is a very small island surrounded by the sea. Its AWS started in the summer of 2000. The nearest stations are Verlegenhuken (180 km WSW) and Ny-Ålesund (320 km SW). Not surprisingly, the uncertainty was smaller when using Verlegenhuken instead of Ny-Ålesund as a predictor. Adding the ice cover over 100 km^2 around Karl XII Island improved the interpolations during the winter months NDJFM. Therefore, a two-predictor approach was adopted.

In the daily series of Karl XII-øya 18 % are missing values. Unfortunately, only 44 % of them could be interpolated by the Verlegenhuken series due to missing values for this predictor. The rest of the interpolations were performed by using Ny-Ålesund instead of Verlegenhuken, whereas the ice cover predictor remained the same. The variance accounted for was smaller and the RMSE larger when using Ny-Ålesund instead of Verlegenhuken. This is shown in **Supplementary Table 2** where the uncertainties of the interpolations are shown both for the interpolations with Verlegenhuken and with Ny-Ålesund as predictors.

Adjustments in the first part of the Verlegenhuken composite series

For interpolations in the Verlegenhuken composite series special precautions were taken. The coefficients in Eqn. 4 were calculated with the data for the period 1996-2020, i.e. the Gråhuken period was omitted. From the theory of regression analysis, we have[10]:

$$D = T - T_{int} = 0 \qquad (5)$$

where D is the mean difference between the observed, T, and interpolated, T_{int}, daily mean temperature in the period 1996-2020.

For the Gråhuken part of the composite series, 1991-1995, we have:

$$D = T - T_{int} = C, \text{ or } T = T_{int} + C \qquad (6)$$

For some months the difference between the observed and the interpolated values was so high that they undoubtedly needed adjustments before they could be adopted into the Verlegenhuken series. It revealed that during the brightest parts of the year, i.e. April to August, $D \approx 2$ °C. This indicates that

the high temperatures might be an overheating problem due to inadequate screening of the temperature sensor for shortwave radiation. Therefore, each month in this season was adjusted in the Gråhuken part of the series, i.e. 1991-1995, see adjustments in **Supplementary Table 2**. The potential monthly adjustments for the dark season, September - March, shifted between negative and positive values. Their mean value was close to zero and no adjustment was performed for those months.

Correlation between SAT from renalyses and instrumental observations

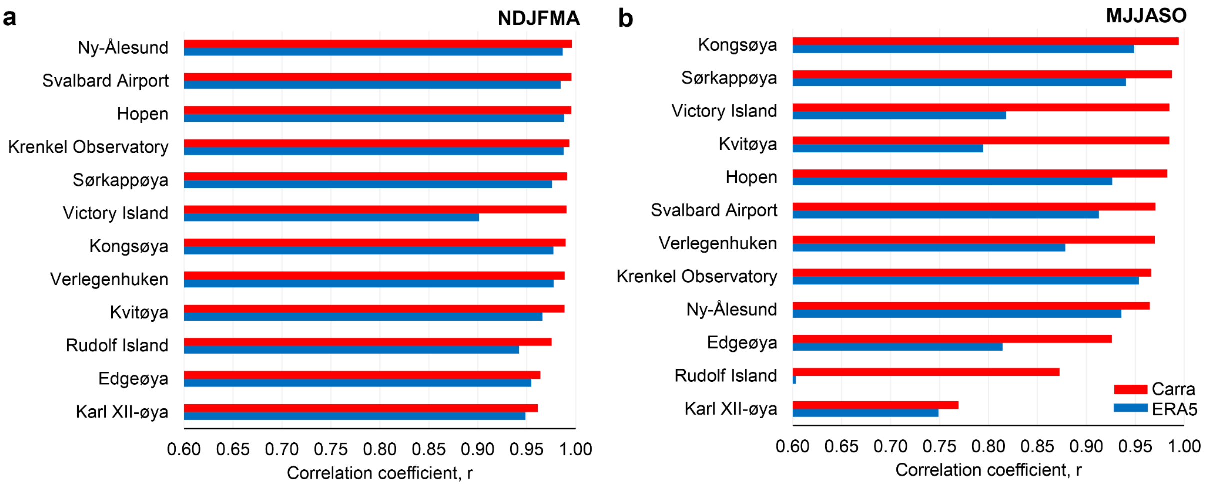

Supplementary Fig. 5 Pearson correlation coefficient *r* between monthly in-situ SAT and SAT for CARRA (red) and ERA5 (blue) for stations included in the study for the period 1991-2020. a Coldest months Nov-Apr (NDJFM) and **b** warmest months May-Oct (MJJASO). The data are ranked according to the highest correlation with CARRA.

SAT biases and Standard Deviation of Error (SDE) for different sea ice conditions

Supplementary Table 3 Summary of SAT biases and Standard Deviation of Error (SDE), for the period 1998-2018, for different sea ice conditions during November to April; "sea ice" (close ice - very close ice, SIC >70%), "mixed ice" (very open ice - open ice, SIC=10-70%) and open water (SIC < 10%). The numbers in parentheses are the SDE normalized with the variability (standard deviation) of the observations and the mean observed SAT for each site and category is given in a separate row.

Sea ice dependence NDJFMA		Close ice		Mixed ice		Open water	
		BIAS	SDE	BIAS	SDE	BIAS	SDE
**Karl XII-øya**	**CARRA**	1.8	4.0 (0.42)	1.4	2.0 (0.31)	0.6	1.1 (0.22)
	**ERA5**	3.5	4.7 (0.49)	1.7	2.2 (0.35)	0.6	1.3 (0.26)
	**SAT**	-19.8		-11.4		-6.3	
**Edgeøya**	**CARRA**	-0.3	2.1 (0.27)	0.1	1.1 (0.23)	0.3	0.5 (0.14)
	**ERA5**	0.5	2.5 (0.33)	0.4	1.4 (0.30)	0.4	0.8 (0.23)
	**SAT**	-13.7		-6.1		-3.9	
**Verlegenhuken**	**CARRA**	0.0	2.4 (0.33)	0.3	1.8 (0.29)	0.6	0.7 (0.15)
	**ERA5**	1.1	2.6 (0.36)	0.5	1.9 (0.32)	0.6	1.1 (0.24)
	**SAT**	-19.2		-13.1		-7.9	
**Ny-Ålesund**	**CARRA**	0.7	1.0 (0.15)	0.8	0.8 (0.13)	0.5	0.6 (0.11)
	**ERA5**	-1.3	1.5 (0.23)	-1.8	1.5 (0.24)	-1.8	1.1 (0.21)
	**SAT**	-14.9		-11.0		-7.3	
**Svalbard Airport**	**CARRA**	1.2	1.4 (0.18)	0.8	1.0 (0.15)	0.5	0.7 (0.12)
	**ERA5**	-1.6	2.7 (0.36)	-2.2	1.9 (0.27)	-2.3	1.4 (0.24)
	**SAT**	-14.2		-11.5		-7.1	
**Kongsøya**	**CARRA**	-0.1	1.5 (0.20)	0.1	0.5 (0.10)	0.3	0.3 (0.09)
	**ERA5**	1.2	2.5 (0.33)	1.3	1.3 (0.26)	1.3	0.9 (0.23)
	**SAT**	-13.3		-8.2		-5.0	
**Kvitøya**	**CARRA**	-0.4	1.2 (0.16)	-0.1	0.8 (0.16)	0.1	0.3 (0.08)
	**ERA5**	0.9	2.3 (0.31)	1.5	1.5 (0.30)	1.4	1.0 (0.23)
	**SAT**	-13.5		-8.9		-6.3	
**Sørkappøya**	**CARRA**	0.3	1.1 (0.20)	1.3	0.9 (0.18)	0.8	0.5 (0.15)
	**ERA5**	2.0	2.0 (0.35)	2.5	1.6 (0.31)	1.2	0.9 (0.25)
	**SAT**	-13.2		-7.9		-2.5	
**Hopen**	**CARRA**	-0.7	1.0 (0.15)	-0.1	0.7 (0.13)	0.1	0.4 (0.12)
	**ERA5**	0.2	1.8 (0.25)	0.7	1.3 (0.23)	0.7	0.8 (0.22)
	**SAT**	-11.5		-6.5		-3.0	
**Krenkel Obs.**	**CARRA**	0.1	1.5 (0.20)	-0.4	1.4 (0.20)	NA	NA
	**ERA5**	-1.0	2.2 (0.3)	-0.8	1.7 (0.25)	NA	NA
	**SAT**	-18.3		-11.4		NA	
Average abs bias (all sites)	***CARRA***	*0.6*	*1.7 (0.22)*	*0.5*	*1.1 (0.19)*	*0.4*	*0.6 (0.12)*
	ERA5	*1.3*	*2.5 (0.33)*	*1.2*	*1.7 (0.28)*	*1.1*	*1.0 (0.23)*
	SAT	*-15.2*		*-9.6*		*-4.9*	

Seasonal trends in surface air temperature compared with reanalyses

-

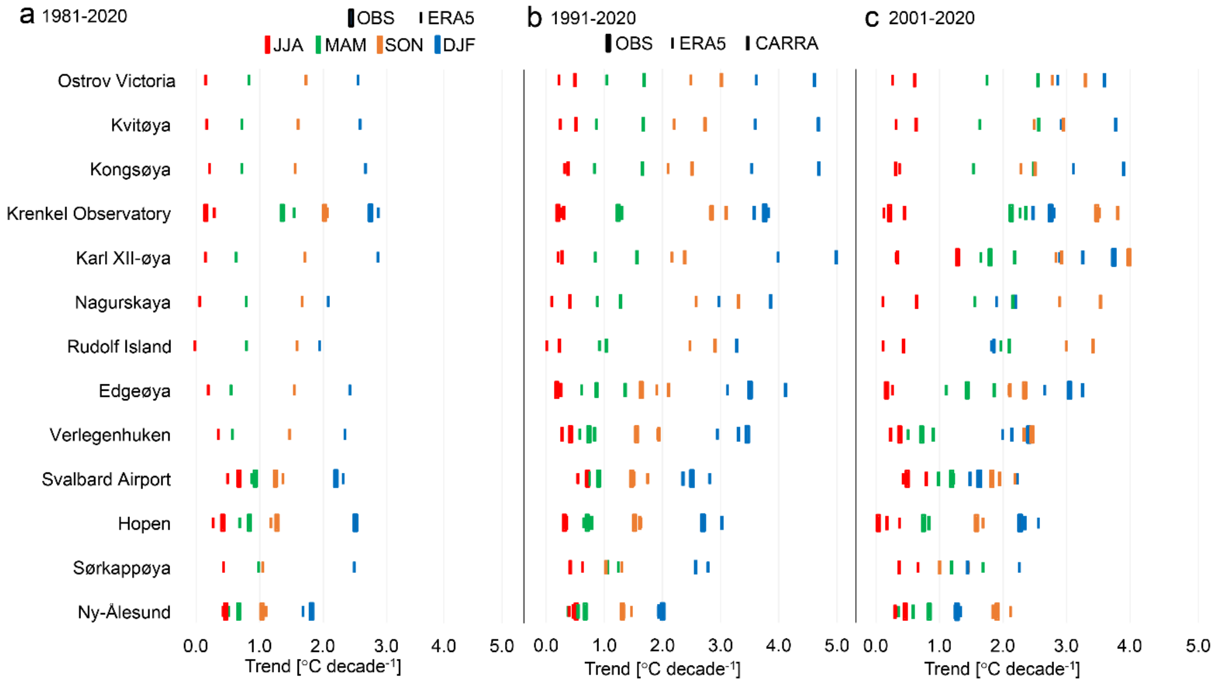

Supplementary Fig. 6 Seasonal trends in surface air temperature compared with reanalyses. Estimated SAT trends from observations (circles), ERA5 (blue bars) and CARRA (red bars, only in b and c) for the three different time periods: **a** 1981-2020, **b** 1991-2020 and **c** 2001-2020. The order of stations follows the ranked annual trends computed from CARRA reanalysis for the period 2001-2020. Similar results but for annual values can be found in Fig. 4.

Monthly trends in sea ice concentration

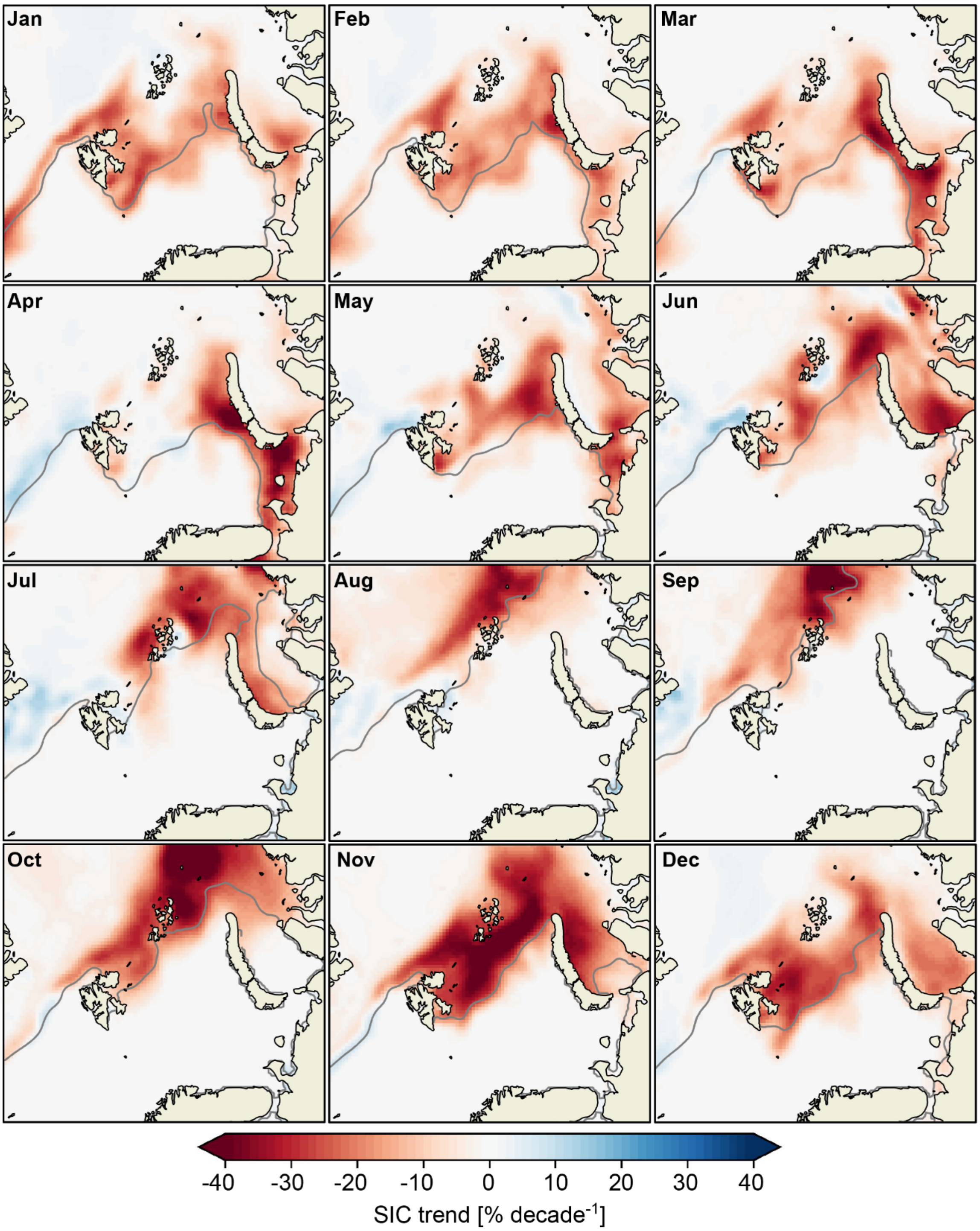

Supplementary Fig. 7 Monthly trends in SIC (%/decade) with mean 15% SIC contour line marked in grey for the period 2001-2020. Based on OSI SAF data. The maps were generated using Python 3.6 (http://www.python.org) including pyresample 1.19 and cartopy 0.18.

Cumulative air temperature anomalies within various large-scale atmospheric circulation types

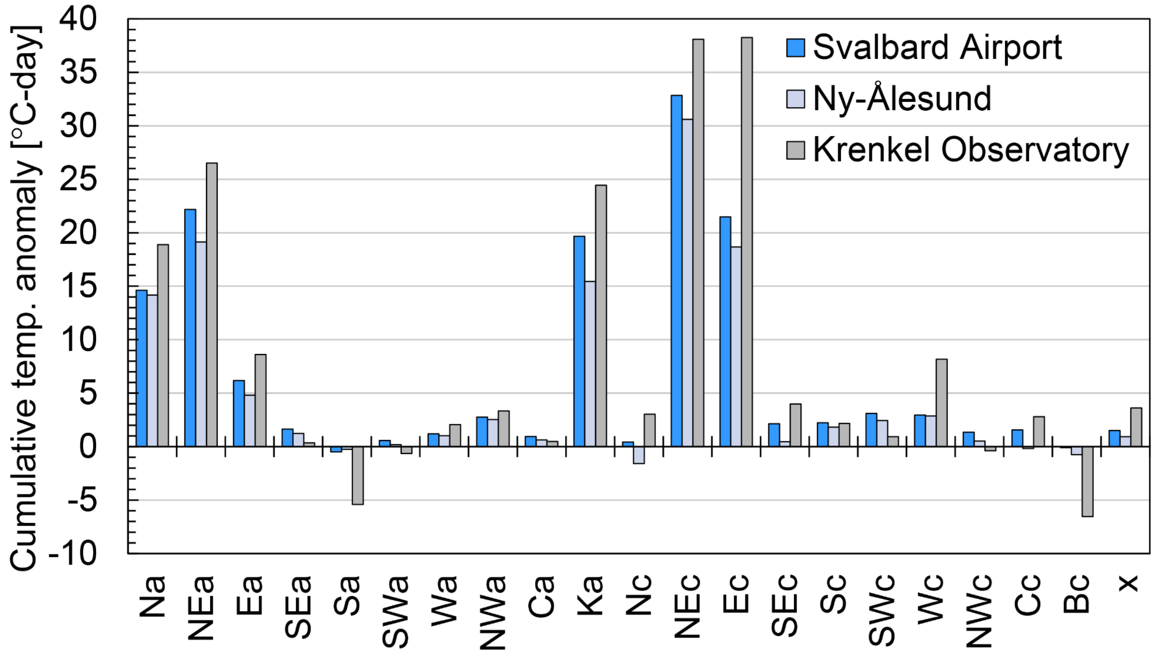

Supplementary Fig. 8 Cumulative air temperature anomalies during the period 2001–2015 (with respect to the 1971–2000 mean) within various large-scale atmospheric circulation types during winter (DJF) for Ny-Ålesund, Svalbard Airport and Krenkel Observatory. The circulation types are denoted by capital letters that show the direction of air advection (e.g., N = northern, NE = northeastern etc) and small letters that show the type of pressure system (a = anticyclone, c = cyclone). In addition to the 16 types with distinct air advection, the dataset includes four nonadvectional types (Ca = anticyclonic center over or very close to Spitsbergen, Ka = anticyclonic ridge, Cc = center of cyclone over or very close to Spitsbergen, and Bc 5 cyclonic trough) and one unclassified type x. The circulation types correspond to the geostrophic wind direction assessed using the sea level pressure pattern over Svalbard. Modified from Isaksen et al.[11], where more details are available.

References

1. Tietjen, G. L. & Moore R. H. Some Grubbs-type statistics for the detection of several outliers. *Technometrics* **14.3**, 583-597 (1972).

2. Ivanov, B. et al. Assessment of long-term changes in the surface air temperature from the High Arctic archipelago Franz Joseph Land from 1929 to the present (2017). *Czech Polar Reports* **11 (1)**, 114-133, https://doi.org/10.5817/CPR2021-1-9 (2021).

3. Førland, E. J., Benestad, R. E., Hanssen-Bauer, I., Haugen, J. E. & Skaugen T. E. Temperature and precipitation development at Svalbard 1900–2100. *Adv. Meteor.* **2011**, 1–14, https://doi.org/10.1155/2011/893790 (2011).

4. Nordli, Ø., Przybylak, R., Ogilvie, A. E. & Isaksen, K. Long-term temperature trends and variability on Spitsbergen: the extended Svalbard Airport temperature series, 1898-2012. *Polar Res.* **33**, https://doi.org/10.3402/polar.v33.21349 (2014).

5. Nordli, Ø. et al. Revisiting the extended Svalbard Airport monthly temperature series, and the compiled corresponding daily series 1898–2018. *Polar Res.* **39**, https://doi.org/10.33265/polar.v39.3614 (2020).

6. Gjelten, H.M. et al. Air temperature variations and gradients along the coast and fjords of western Spitsbergen. *Polar Res.* **35**, https://doi.org/10.3402/polar.v35.29878 (2016).

7. Hanssen-Bauer, I. et al. (eds.). *Climate in Svalbard 2100 – a knowledge base for climate adaptation*. NCCS report 1/2019, ISSN 2387-3027 (2019).

8. Mestre O. et al. HOMER: a homogenization software - methods and applications. *Idojaras-Q. J. Hungarian Meteorol. Service* **117**, 47-67 (2013).

9. Renner, A. H. H. et al. Variability and redistribution of heat in the Atlantic Water boundary current north of Svalbard. *J. Geophys. Res.-Oceans* **123**, 6373– 6391, https://doi.org/10.1029/2018JC013814 (2018).

10. Wilks, D.S. Statistical Methods in the Atmospheric Science. An Introduction. *International Geophysics series*, **59**, Academic Press, 159-181 (1995).

11. Isaksen, K. et al. Recent warming on Spitsbergen - Influence of atmospheric circulation and sea ice cover. *J. Geophys. Res. Atmos.* **121**, 11913– 11931, https://doi.org/10.1002/2016JD025606 (2016).
